# Paracoccidioidomycosis: Current Perspectives from Brazil

**DOI:** 10.2174/1874285801711010224

**Published:** 2017-10-31

**Authors:** Rinaldo Poncio Mendes, Ricardo de Souza Cavalcante, Sílvio Alencar Marques, Mariângela Esther Alencar Marques, James Venturini, Tatiane Fernanda Sylvestre, Anamaria Mello Miranda Paniago, Ana Carla Pereira, Julhiany de Fátima da Silva, Alexandre Todorovic Fabro, Sandra de Moraes Gimenes Bosco, Eduardo Bagagli, Rosane Christine Hahn, Adriele Dandara Levorato

**Affiliations:** 1 Department of Tropical Diseases, Faculdade de Medicina de Botucatu – São Paulo State University – UNESP, São Paulo, Brazil; 2 Department of Dermatology, Faculdade de Medicina de Botucatu – São Paulo State University – UNESP, São Paulo, Brazil; 3 Department of Pathology, Faculdade de Medicina de Botucatu – São Paulo State University – UNESP, São Paulo, Brazil; 4 Laboratory of Experimental Immunology, Department of Biological Science, Faculty of Science, São Paulo State University – UNESP, São Paulo, Brazil; 5 Department of Infectious and Parasitic Diseases, Faculdade de Medicina – Federal University of Mato Grosso do Sul – UFMS, Brazil; 6 Instituto Lauro de Souza Lima, São Paulo State, Bauru, Brazil; 7 Unit of Experimental Research, Faculdade de Medicina de Botucatu – São Paulo State University – UNESP, São Paulo, Brazil; 8 Department of Microbiology and Immunology – Instituto de Biociências de Botucatu – São Paulo State University – UNESP, São Paulo, Brazil; 9 Laboratory of Investigation and Mycology, Federal University of Mato Grosso, Faculty of Medicine Cuiabá, Mato Grosso, Brazil

**Keywords:** Paracoccidioidmycosis, *Paracoccidioides brasiliensis*, *Paracoccidioides lutzii*, Endemic mycosis, Systemic mycosis, Antifungal compounds

## Abstract

**Background:**

This review article summarizes and updates the knowledge on paracoccidioidomycosis. *P lutzii* and the cryptic species of *P. brasiliensis* and their geographical distribution in Latin America, explaining the difficulties observed in the serological diagnosis.

**Objectives:**

Emphasis has been placed on some genetic factors as predisposing condition for paracoccidioidomycosis. Veterinary aspects were focused, showing the wide distribution of infection among animals. The cell-mediated immunity was better characterized, incorporating the recent findings.

**Methods:**

Serological methods for diagnosis were also compared for their parameters of accuracy, including the analysis of relapse.

**Results:**

Clinical forms have been better classified in order to include the pictures less frequently observesiod.

**Conclusion:**

Itraconazole and the trimethoprim-sulfamethoxazole combination was compared regarding efficacy, effectiveness and safety, demonstrating that azole should be the first choice in the treatment of paracoccidioidomycosis.

## 1 INTRODUCTION

### 1.1 Definition

Paracoccidioidomycosis (PCM) is a systemic granulomatous disease that can affect any organ in the body, predominantly the lungs, organs rich in mononuclear phagocyte system cells, the mucous membrane of the upper aerodigestive tract (UADT), the skin and adrenal glands. This condition is caused by thermally dimorphic fungi of the *Paracoccidioides brasiliensis* complex – *P. brasiliensis, P. lutzii* (Pb01) and Pb01-like species. It is an endemic disease limited to Latin America, from Mexico to Argentina.

As the recognition of *P. lutzii* is recent, most of knowledge about the fungus and its interaction with the host has been based on studies with *P. brasiliensis*. For this reason, this article brings a greater number of references related to *P. brasiliensis.*

### 1.2 Brief History

The two earliest cases of PCM were reported in 1908 by Adolpho Lutz [1], who described the clinical manifestations and anatomopathological findings of the disease and isolated its aetiological agent in pure cultures. Lutz also infected guinea pigs, observed the occurrence of thermal dimorphism – a yeast-like phase in tissues and a filamentous phase in culture media – and reproduction by multiple budding. Lutz named the disease pseudococcidial hyphoblastomycosis to distinguish it from coccidioidomycosis, which is caused by *Coccidioides *spp, as well as from Gilchrist’s disease, currently known as blastomycosis and caused by *Blastomyces dermatitidis*.

Despite his major contribution to the understanding of PCM, Lutz did not suggest a name for its aetiological agent. In 1912, Splendore classified the organism as yeast from genus *Zymonema* [2]. In 1928, Almeida and Lacaz introduced the name *Paracoccidioides,* and Almeida named the fungus *Paracoccidioides brasiliensis* in 1930 [3].

Although the disease was given countless names, the one most widely employed to identify Lutz’s mycosis was South American blastomycosis. However, reports of autochthonous cases from Central America and Mexico showed that it was not restricted to South America and (together with) the trend to integrate the name of the disease with the name of its aetiological agent, *Paracoccidioides brasiliensis*, led to the preferential use of the term paracoccidioidomycosis, which was suggested by Jordan in 1946 [4] and officialised at the Medellin Symposium (Colombia) [5].

Reports of many cases of PCM presenting with lesions in the mucous membrane of the UADT led to consideration of this area as the entry point for *P. brasiliensis* into the body. However, in 1956 Gonzalez-Ochoa suggested that the lungs are actually the entry point [6], a hypothesis that was reinforced by Mackinnon’s findings in an experimental model [7]. The existence of a PCM primary complex was subsequently confirmed by Severo *et al* [8]. The existence of many individuals with *Paracoccidioides* infection was revealed by Fonseca Filho and Arêa Leão [9] through an intradermal reaction induced using a *P. brasiliensis* culture filtrate as antigen. This antigen was termed paracoccidioidin [10]. Considering the lungs as the portal of entry for *P. brasiliensis* into the organism, the fungus could be isolated in the saprophyte state from nature and could live inside a heterothermic organism native to endemic areas [11]. Indeed, isolation from the soil was achieved by Albornoz [12] and from armadillos by Naiff *et al* [13].

The histopathological characteristics of PCM were thoroughly investigated by Cunha Motta in patients with lesions affecting organs that are rich in mononuclear phagocyte system cells [14]. In turn, Fialho [15] demonstrated that lung involvement was very frequent and made an accurate characterisation of it. The correlation between histopathological findings and cell-mediated and humoral immunity was established at the School of Medicine of Botucatu [16].

*P. brasiliensis* exhibits a complex antigenic structure that includes glycoproteins, glycopeptides, lipids and polysaccharides. The correlation between virulence and presence of α-1,3-glucan in the cell wall was the point of departure for various studies of the biochemistry and dimorphism of the fungus [17]. Arc E, detected by Yazarbal via immunoelectrophoresis [18], revealed the presence of specific serum antibodies against the 43-kDa glycoprotein. This protein constitutes the dominant antigen of *P. brasiliensis* and was later characterised by Puccia *et al* [19].

The serological assessment of patients with PCM was first performed by Moses [20] using the complement fixation and precipitation tests, which were later standardised by Fava-Netto using a polysaccharide antigen [21, 22]. Next, Restrepo introduced the double agar gel immunodiffusion test (DID). This test was found to be simple to perform, to be highly specific and to be useful for the follow-up of patients undergoing treatment [23]. Subsequently, Biagione *et al* [24]. found a correlation between the serum levels of antibodies on the DID test and PCM severity.

The **in vitro** conversion of the mycelial to the yeast-like phase, which confirmed Lutz’s original observation (mycelial phase **in vitro** and yeast-like phase in guinea pigs) was demonstrated by Negroni [25] and was introduced into the laboratory routine for the identification of *P. brasiliensis*. Fluorescein-linked immunoglobulin conjugates were also added to the techniques used for the identification of *P. brasiliensis* in clinical samples [26].

The depression of the cell-mediated immune response in patients with PCM was demonstrated by Mendes & Rafael [27] and Musatti *et al* [28]. This effect was followed by reports that indicated a correlation between depression of cell-mediated immunity and patient severity [29] and that immunosuppression is antigen-dependent [30].

In PCM, the various possible outcomes of the host-parasite interaction – infection only, mild, moderate or severe clinical forms – as well as hormonal influences point to the relevance of the genetic background for the development of disease. The line of research developed by Calich *et al*. with isogenic mice that were susceptible and resistant to *Paracoccidioides* infection [31] has greatly contributed to the understanding of PCM immunopathology.

In 1940, the use of sulphapyridine by Oliveira Ribeiro was found to be an efficacious drug for the treatment of PCM [32]. The second therapeutic agent, amphotericin B, an antifungal from another chemical class, was introduced only 18 years later by Lacaz & Sampaio [33]. These two medications represented a revolution in the prognosis of PCM.

Studies on the phylogeny [34] and genomics [35] of PCM-causing fungi allowed the demonstration of more than one species in the genus *Paracoccidioides*, with variation in their geographical distribution.

Despite involving individuals joined in agribusiness, base of the Brazilian economy, PCM still remains a neglected disease due to the following characteristics: it occurs in poor and rural environments; disproportionately affects low-income populations; perpetuates a vicious cycle of the disease, between poverty and inadequate health care; does not receive attention from the developed world; is outside the purview of the Global Fund and its related programs; promotes poverty by causing long-lasting sequelae and devastating impacts on individual work productivity and quality of life; generally disables, rather than kills; involves patients who are not able to obtain the drug therapy; and, finally, it affects patients who frequently ask for medical care very late, when the disease is at an advanced stage. In addition, like several neglected tropical diseases, PCM has been extensively studied by researchers from developed countries or from renowned research centers in developing countries [36, 37].

Surveillance and control programs constitute the first step to change this condition, but compulsory notification was not implemented in Brazil, in spite of the numerous efforts of the Secretaria de Vigilância em Saúde, Ministério da Saúde (SVS-MS) [36]. This program was developed as an initiative of some states of the federation [36].

## 2 ETIOLOGY

### 2.1 Mycology

**Fig. (1):**
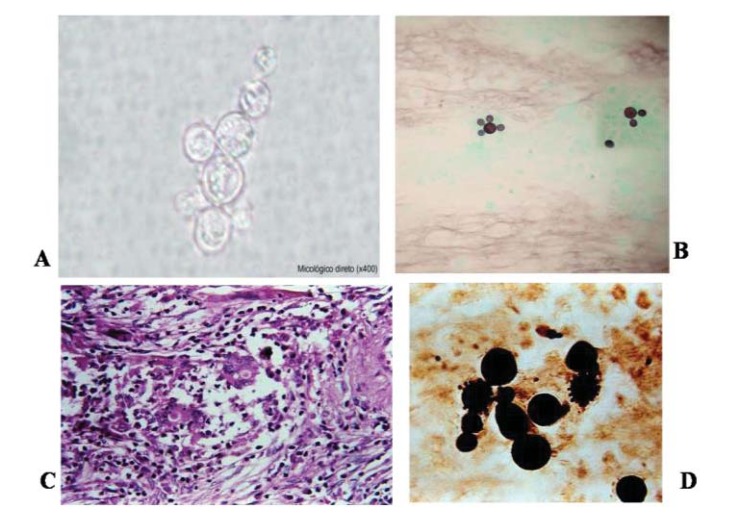
*Paracoccidioides brasiliensis.* A – a 10% KOH preparation of sputum showing multiple budding yeast forms, with birefringent walls; B – cell-block preparation of sputum (GMS, x400) showing the mickey-mouse and pilot will; C – histopathological examination showing granulomatous lesions with fungi within multinucleated giant cells (HE, x160); D – histopathological examination showing multiple budding yeast form (GMS x160). [B,C,D courtesy of the Department of Pathology – Faculdade de Medicina de Botucatu – UNESP].

Fungi of the *Paracoccidioides* genus are thermally dimorphic, and can be cultivated as mycelium or yeast cells. Cultivated at 25^o^C, after 15 to 30 days, a white colony is observed, becoming velvety and brownish. By using agar Sabouraud dextrose, it is possible to observe septated hyaline hyphae, with branches; in this culture medium the production of conidia is rare. When cultivated in media without carbohydrates but with natural substrates, arthroconidia, aleuroconida and arthroaleuroconidia present 2 to 5µm in diameter. At 37^o^C and in human and animal tissues, *P. brasiliensis* resembles yeast cells. Its growth is slow, showing rugged and pleated colonies, from 7 to 20 days after inoculation. Under direct microscopy, the yeastlike cells vary in size and shape, being oval, spherical or eliptical, with birefringent walls. The mother-cells present 20 to 30µm in diameter and can produce 10 to 12 daughter-cells, with 2 to 10µm, forming the characteristic *pilot wheel*. The mother-cell with two daughter-cells frequently resembles the *mickey-mouse* (Figs. **1A**, **B**, **D**).

Its observation is easier when stained with lactophenol cotton blue, Gomori methanamine silver (GMS) and periodic acid of Schiff (PAS) than with hematoxilin-eosin (HE), which hardly stain the fungal wall. On the other hand, HE stain permits the identification of the preserved fungal cells.

### 2.2 Phylogeny

The high prevalence of negative serological tests in mycologically confirmed patients from the Midwest Region using antigens from isolates in the Southwest Region (B-339) suggested genetic differences among samples from distinct origins [38]. In addition, genetic differences demonstrated by RAPD were detected in fungal cells isolated from the forearm and face of the same patient, at the same admission [39]. Then, an isolate from a patient of the State of Mato Grosso (Midwest Region, Brazil) was well characterized and, due to the differences of *P. brasiliensis*, it was coined isolates 550B and 550F. Moreover, it was demonstrated that all the isolates from Mato Grosso (Midwest Region, Brazil) belonged to the cluster Pb01-like [40]. The study of these and other isolates led to the description of a new species of *Paracoccidioides*, initially coined Pb-01, which received the name of *Paracoccidioides lutzii* in honour to the researcher who reported the first two cases of PCM [41]. In the same study, the cryptic species S_1_, PS_2_ and PS_3_ were molecularly characterized.

### 2.3 Transition from the Mycelial to the Yeast Phase

Species of the *P. brasiliensis* complex have the ability to change their morphology from a multicellular filamentous form to a unicellular form when they infect host tissues, a process referred to as dimorphism [42]. The temperature is the only factor triggering *P. brasiliensis* dimorphism, when α-1,3-glucan, the major cell wall neutral polysaccharide constituent of the pathogenic yeast phase replaces almost entirely the β-glucan, that comprises the neutral polysaccharide of the vegetative mycelial phase. This transition behaves as a mechanism of escape and α-1,3-glucan like a virulence factor.

## 3 ECOLOGY

### 3.1 Ecological Aspects of *P. Brasiliensis* and *P. Lutzii*

Despite important advances in knowledge of the biology of the etiological agent(s) of paracoccidioidomycosis (PCM), we are far from having the complete picture of relevant biological factors, such as the ecology of these agents. Few reports are present in the existing literature that address the isolation of these pathogens from the environment [43-47], and while fungal isolation from the faeces of bats and penguins and dog food contaminated with soil has been reported, only casual remarks with limited reproducibility have been made [48-51]. The lack of outbreaks and prolonged latency period of the disease, together with human migration, have resulted in the exact infection source remaining unknown [45]. An important clue for ecological studies on *P. brasiliensis* was the finding of naturally infected nine-banded armadillo (*Dasypusn ovemcinctus*) in endemic areas [13, 52-55]. The fungus was also isolated from another armadillo species, *Cabassous centralis*, reinforcing that armadillos are in constant contact with the pathogen in the environment [56]. The systematic recovery of *P. brasiliensis* from armadillo tissues has demonstrated the importance of this animal in PCM endemic areas, helping locate hot spots of the fungus occurrence in some environments, such as in some restricted and/or protected soil conditions, in places containing natural and anthropic disturbed vegetation, near water sources [53, 57]. The environment represented by the armadillo burrow and its surroundings, associated with biotic and abiotic features, may contribute to the development of the fungus saprobic stage in nature, as already demonstrated by Terçarioli *et al* [58]. However, *P. lutzii* has not previously been isolated from armadillos, even when the animals were captured in the central or western regions of Brazil, which have been identified as endemic areas for this species [59]. On the other hand, regardless of whether *P. lutzii* has been isolated from armadillo tissues, both *P. brasiliensis* and *P. lutzii* have been molecularly detected in soil and aerosol samples obtained from armadillo burrows and habitats [59].

Phylogenetic studies have suggested that *P. brasiliensis, P. lutzi*, and other Onygenalean (Ascomycota) dimorphic fungi, such as *Blastomyces dermatitidis*, *Histoplasma capsulatum*, *Coccidioides immitis* and *C. posadasii*, have evolved in association with animal hosts since ancient times, adapting to two distinct ecological niches: the first represented by natural saprobic conditions in soil and the second represented by the live tissues of animal hosts [57, 60, 61]. This co evolution with animals may have induced irreversible genetic changes in these pathogens, resulting in increased adaptation to biotrophic lifestyles and significant reductions in their saprobic forms. Though it is clear that saprobic forms of *P.brasiliensis* and *P.lutzi* persist in the environment, producing infective propagules (conidia) that can induce primary infection of the lung via the airborne route, it is also quite certain that the saprobic phase may be relatively transitory and occur under only special environmental conditions, such as below soil surfaces, in burrows and other similarly protected habitats [57].

Although there are several indications that *P. brasiliensis* and *P. lutzii* exhibit different geographical distributions, this subject is far from resolved. While *P. lutzii* isolates have been detected mainly in the central and western regions of Brazil, this species has previously been isolated from one patient and detected molecularly in aerosol samples from the southeast region [59, 62, 63]. Experimental studies have suggested that the ability to produce infective conidia differs between genetic groups or cryptic species of *Paracoccidioides*, which, in turn, may determine the incidence of infection. For example, the S1 and PS2 genotypes occur sympatrically in Southeast Brazil at a rate of 9:1 (S1:PS2), and conidia production has been identified to be higher in S1 than PS2 isolates under laboratory conditions [64].

Studies were carried out in PCM patients with the acute/subacute form evaluating the incidence of the cases in relationship with several climatic conections – precipitation, air temperature, absolute and relative humidity, soil water storage, and Southern Oscillation Index – SOI [65]. The results suggest that higher water storage two years before exposure may explain the relationship between *P. brasiliensis* and rainfall/humidity. Probably, there is fungal growth after increase in soil water storage in the long term followed by greater spore release with increase in absolute air humidity in the short term. There are indirect evidences that *P. brasiliensis* grows preferentially 2-20cm below the soil suface, a condition that protects from the competitor, abundant in the first soil layers. Human rural activities remove the level surface of the soil, exposing the filamentous, spore producing form, of the fungus. If the absolute humidity is high enough at that moment, the conidia and fragments of mycelia are aerosolized.

It was also described the first well-documented cluster of cases of the acute/subacute form of PCM, with potential relationship with the El Niño Southern Oscillation (ENSO), phase in 1982-1983 [66]. The ENSO behaviour explains the rainfall variability not only in the Brazilian equatorial region, but also in the Southern. The atypically high soil water storage in 1982-1983 due to precipitations higher than two standards deviations above the mean in the Botucatu region (São Paulo state), area of this study, can be implicated in this finding.

## 4 VETERINARY ASPECTS

### 4.1 Infection of *Paracoccidioides Brasiliensis* in Domestic and Wild Animals

Studies of natural infection with *P. brasiliensis* in animals are relatively scarce when compared with studies conducted on human populations. Evaluations of fungal infections in animals have been mostly performed using intradermal tests, serological surveys, histopathological analyses, molecular tools and isolation of the pathogen in culture. Most of these studies have been carried out in South and Southeast Brazil, which are areas of high PCM endemicity.

Delayed hypersensitivity tests performed using paracoccidioidin as the antigen under study on wild terrestrial (coatimundis and *Felidae* species) and arboreal animals (weeping-capuchins and marmosets) have demonstrated a significantly higher rate of positivity in terrestrial (83%) than arboreal animals (22%) [67]. Among domestic animals, positivity rates have been reported to be higher in equines (64%) than sheep (41%) and cattle (40%) [68].

While serological surveys have been performed on different animal species, they have predominantly been carried out on dogs. In these studies, the observed rates of positivity were 78% in dogs from Botucatu (São Paulo State, Brazil); 74% in dogs from São Paulo (São Paulo State, Brazil) [69]; and 90%, 49% and 15% in dogs from rural, suburban and urban areas of Londrina (Paraná State, Brazil), respectively [70].

Infection of animals by *P. lutzii* was observed in the State of Rio Grande do Sul – Brazil [71]. The data showed no difference of prevalence between infection by *P. brasiliensis* and by *P. lutzii*. Comparison among animals revealed increased prevalence of infection by *P. brasiliensis* and *P. lutzii* in dogs and by both species in wild animals. In addition, the prevalence of serum positivity varied regarding the geographic origin of the animals.

Dogs are susceptible to experimental *Paracoccidioides* infection, as was demonstrated by the infection of four puppies [72]. Two of the puppies died one week after inoculation and were autopsied. Histopathological examination showed granulomas in the lungs, spleen and liver, and the fungus was recovered in culture. The remaining two dogs were evaluated one and five months post-inoculation; no gross lesions were identified in the organs of these puppies, and the fungus was not recoverable.

Natural disease in dogs was first reported in a female adult Doberman Pinscher from Mogi Guaçu (São Paulo State, Brazil), which exhibited poor general condition and cervical lymph node enlargement [73]. The lymph nodes of this dog were biopsied, and the histopathological examination revealed active PCM with numerous typical *P. brasiliensis* yeast forms. After treatment with ketoconazole, total regression of the lymphadenomegaly was observed; however, clinical recurrence was observed 18 months later, and the dog was euthanized but not autopsied. The second case was reported in a female adult Doberman Pinscher from Curitiba (Paraná State, Brazil) [74]. This dog presented emaciation, generalized lymphadenomegaly and hepatosplenomegaly. The popliteal lymph node of the dog was biopsied and cultured. *P. brasiliensis* was recovered, and the animal was successfully treated with itraconazol for two years.

Molecular tools have facilitated the detection of *P. brasiliensis* DNA in both soil and animal tissue samples [72, 73]. Using this approach, a variety of wild animal species that were road-killed in PCM endemic areas in São Paulo State (Brazil) were found to be infected with *P. brasiliensis*, including two species of armadillos (*Dasypus novemcinctus* and *Dasypus septemcinctus*) and guinea pig (*Cavia aperea*), raccoon (*Procyon cancrivorus*), porcupine (*Sphiggurus spinosus*) and tayra (*Eira barbara*) species [75].

The discovery of nine-banded armadillos (*Dasypus novemcinctus*) naturally infected by *P. brasiliensis* constituted a landmark finding in the ecological study of this pathogen, leading to a better understanding of its possible distribution in nature [13, 52, 53]. *Cabassous centralis* (naked-tailed armadillo) has also been found to be naturally infected in Colombia, as was demonstrated for the first time by isolating this fungus from armadillo tissues and confirming its identification by sequencing the ITS and gp43 regions [56].

Armadillos belong to the order Xenarthra, an ancient order of mammals that also comprises sloths and anteaters. It is believed that this order has existed in South America for 65 million years, and because the order Onygenales originated 150 million years ago, it has been proposed that the two orders co-evolved in concert [60]. Of the xenarthrans, armadillos are most important in the ecology of *P. brasiliensis*. Paracoccidioidal infections in two species of anteater (*Tamandua tetradactyla* and *Myrmecophaga tridactyla*) were reported for the first time in São Paulo State (Brazil) and detected by molecular amplification of the ITS region, through which the fungus was identified in the lungs, liver, spleen and mesenteric lymph nodes of each animal [76]. The final link in the epidemiological chain connecting *P. brasiliensis* and xenarthran species was formed by identification of naturally acquired PCM disease in a two-toed sloth (*Choloepus didactylus*) from Central America. The animal was from French Guiana and died during the quarantine period in a pet shop at Monterrey (Mexico). The diagnosis was confirmed by the presence of granulomatous lesions in the lungs, liver, spleen and kidneys, which exhibited typical multi budding *P. brasiliensis* yeasts [77].

These findings show that *P. brasiliensis* and *P. lutzii* infection in animals from areas endemic to the human disease is more common than previously realized; thus, it is important that veterinarians be aware of the potential for *Paracoccidioides* infection.

## 5 EPIDEMIOLOGY

The occurrence of PCM has been reported from Mexico to Argentina, with the largest number of patients being from five countries: Brazil, Venezuela, Colombia, Ecuador and Argentina. Chile is the single South American country without any reported autochthonous case, which is due to climactic conditions that are unfavorable for the fungus’ survival in the soil. In Brazil, the Southeast, Centre-West and South regions have the largest number of cases.

PCM affects current or former rural workers who are exposed to intense and continued contact with the soil. The disease predominates among males due to the protection conferred by estrogen, which inhibits or hinders the transformation of conidia and mycelial fragments into the yeast-like form, which is pathogenic [78-80]. The epidemiological pressure associated with the predominance of disease among rural male workers can also influence the distribution of cases per gender. The male:female ratio is 1.7:1.0 among patients with the acute/subacute form (AF) and 22.0:1.0 among those with the chronic form (CF) of the disease. PCM predominates among individuals aged 30 to 59 years of age and also among those of mixed race [81]. The Infectious Diseases Service of the School of Medicine of Botucatu receives on average 15 new cases of PCM per year [82].

Skin tests revealed high rates of infection in several Brazilian areas, with no difference as to gender. The Botucatu region (São Paulo state) is hyperendemic and the paracoccidioidal infection can occur at an early age, including in 5-year-old children [83].

The use of a specific antigen in the skin tests reduces the possibility of cross reactions with other fungal infections. The gp43 was used as antigen in a survey performed in individuals from rural settlements in a Brazilian Midwest region, demonstrating a prevalence of 45.8% of infection [82].

A study that analysed death certificates detected a greater number of deaths by PCM in the Southeast, Centre-West and South regions of Brazil, but higher mortality rates in the Centre-West region and the state of Santa Catarina [84]. Most deaths correspond to patients over 60 years of age in every region. That study also revealed that PCM is the eighth cause of death among the predominantly chronic or relapsing infectious diseases. The mortality rate in the state of Paraná is 3.48 cases/1 million inhabitants [85]. In the state of São Paulo, the mortality rate is 2.66, 1.58 as primary and1.08 as an associated cause of death. However, in the Botucatu area, which is in the centre-west region of the state of São Paulo, the corresponding rates are 8.73, 4.89 and 3.84, confirming its status as hyperendemic area [86].

A study on the prevalence of blood types among patients with PCM as a function of the severity of disease and compared to healthy subjects suggested that the red blood cell antigens Jka, Jkb, Fyb and Leb might play a role in the immunopathology of disease, possibly as resistance factors [87].

## 6 GENETIC ASPECTS

Infectious diseases are complex traits since acquired and genetic factors related to the host, pathogen characteristics, and environmental conditions contribute to the outcome risk.

The finding that only 2% of individuals exposed to species of *Paracoccidioides* develop PCM [88] favours the hypothesis that a genetic component plays a role in the balance between infection and disease.

The importance of the host genetics in PCM was demonstrated by experimental models using resistant and susceptible mice. The pattern of lesions showed remarkable differences, specially related to the extracellular matrix of the granulomatous lesions. Resistant mice revealed the coexistence of two types of lesions: one type presenting encapsulated nodules constituted mainly of type I collagen, and abundant neutrophil burden in areas of massive fungal destruction; the other type showed residual characteristics, with sparse collagen deposits containing xantomatous-like macrophages, with degenerated fungi. On the other hand, susceptible mice showed only one type of lesion, with less tendency to encapsulation, presence of reticular type III collagen, many plasmocytes, and large number of budding yeasts, with no evidence of fungal destruction [89]. In addition, in the same experimental model, the early influx of neutrophils to the lungs was higher in susceptible rather than in resistant mice infected with *P. brasiliensis* while neutrophil depletion resulted in decreased survival time of susceptible but not resistant mice, showing the influence of genetic factors on the immunoprotective and immunoregulatory role of these cells in PCM [90].

Data on human genetic susceptibility for PCM are scarce and no result was replicated or validated. Some candidate genes have been already tested but the short number of individuals evaluated has been a limitation for these studies. In addition, the selection of controls for studies on genetic association is a critical point, and the exposure to the pathogen should be taken into consideration. Thus, the ideal controls for PCM studies are individuals living and working in rural areas of risk for a long time. Positive intradermal test, performed in healthy individuals with specific antigens, should be used as criteria for control selection since this test has been a good marker to confirm a previous fungal exposure [91].

The occurrence of a disseminated form of PCM in a carrier of primary immunodeficiency related to the IL-12/IL-23-IFN-γ axis also suggests a role of the host genetics in PCM [92]. These disorders are genetic defects which predispose to severe forms of some infections, mainly caused by non-pathogenic mycobacteria, known as Mendelian susceptibility for mycobacterial diseases (MSMD). Thus, genes related to this pathway are candidates to association with susceptibility for PCM. Variants at IFNG and IL12B genes were not associated with clinical forms of PCM in Brazilian patients, while IL12RB1 presented one polymorphism associated with the disseminated chronic form [93]. However, more variants at these genes should be tested in order to elucidate its participation in determining the clinical form.

Polymorphisms at IL4 gene were associated with susceptibility for PCM and with the production of the cytokine by PBMC after specific stimulus [94, 95]. A polymorphism located at the promoter region of IL10 was also associated with PCM in a specific Brazilian population [96]. Variants at CTLA4, TNF and IFNG genes were not associated with PCM [94, 96, 97], but class I and class II HLA alleles were associated with PCM. The first studies demonstrated a higher frequency of HLA-A9 e HLA-B13 alleles in Colombian patients [98]. In Brazilians, B40, Cw1, A2, B7 and B21 alleles had higher frequencies in PCM patients [99, 100]. In another Brazilian study the DR-B1*11 allele was associated with the unifocal chronic form of the disease [101]. These data should be carefully evaluated because of the ethnic differences of these patients as to the region they are from.

Since the clinical forms of PCM are dependent on the host adaptive immune response, the genes driving such response are strong candidates to determine a specific clinical form. Thus, data from patients with the AF and the CF should be analyzed separately. Large-scale genetic studies in PCM are required to elucidate the architecture of the disease aiming better strategies of control, diagnosis and treatment.

Smoking, which is highly common among patients with PCM, increases the risk of pulmonary PCM 10-fold and reduces by 8 years the patients’ age at the onset of symptoms [87].

An evaluation using multilocus sequencing studies, maximum likelihood and Bayesan analyses, carried out on isolates within the *Paracoccidioides* genus, confirmed the existence of two distinct species - *P. brasiliensis* and P. *lutzii* and supported that *P. brasiliensis* isolates are clustered into five distinct lineages - S1a, S1b, PS2, PS3, and PS4. The S1b lineage includes the reference strain Pb18 and the S1a lineage is split into two subclades. PS3 includes isolates from Colombia and Pb 339, an isolate from southeast Brazil, used in the preparation of antigen for diagnostic tests. The Fig. (**2**). shows the phylogeny and recombination in *Paracoccidioides*, and the regional distribution of the isolates in South America. The clinical impact of these findings are related to the preparation of antigens for serological diagnosis and follow-up because the sensitivity of these tests is higher when a specific preparation is used. In addition, clinical manifestations, radiological findings and response to treatment should be evaluated regarding to the *P. brasiliensis* lineage [102].

## 7 PATHOGENESIS

### 7.1 Natural History

The lungs are the usual portal of entry for *Paracoccidioides* sp into the human body; spores reach the terminal bronchioles and alveoli, causing areas of pneumonitis. From these areas, the fungus spreads by the lymphatics to the paratracheal and parabronchial lymph nodes, where it triggers a granulomatous reaction Fig. (**3**). The areas with pneumonitis constitute the parenchymal pole of the paracoccidioidal infection, and the affected regional lymph nodes constitute the lymphatic pole. Together, the parenchymal pole, ascending lymphangitis and satellite lymph node affection is known as PCM primary complex [8, 103].

The host’s immune response to infection with *Paracoccidioides* sp determines the progression of the host-parasite interaction. When the immune response is satisfactory, the body blocks infection at the level of the primary complex and its eventual metastases. In such cases, the inflammatory reaction recedes and scars are formed, which may be sterile or contain viable, albeit latent fungi. These patients exhibit infection only, which is detectable through a positive intradermal reaction to paracoccidioidin. Depending on the balance between the host, parasite and environment, the fungi may remain latent for many years, occasionally for life. However, after a variable, usually long period of time, any imbalance between these factors may result in reactivation of latent foci, a phenomenon known as endogenous reinfection, which triggers disease.

Because a large portion of patients remain in continuous contact with the soil after the initial exposure to the fungus, it is difficult to assess the contribution of a new infection, *i.e.*, the so-called exogenous reinfection, to the triggering of disease. In turn, the development of disease in patients many years after having moved away from an endemic area confirms the relevance of endogenous reinfection.

When the immune response is insufficient at the time of primary complex formation, the fungi multiply and spread first by the lymphatic system and then the haematogenous route to various organs and systems. In such cases, the disease manifests immediately following infection, *i.e.*, after the first contact with the fungus.

PCM (disease) might progress into death or cure. Cure is associated with scar formation in the affected organs, which may eventually cause sequelae, among which lung fibrosis and emphysema with consequent functional impairment stands out. The scars may be sterile or contain viable fungi, which might cause relapse.

Although rare, cutaneous inoculation can also be a portal of entry for *Paracoccidioides* sp. However, to establish that a skin lesion was caused by direct inoculation, the occurrence of local trauma in the affected area two to three weeks before the appearance of regional lymphadenopathy should be confirmed and lung compromise ruled out. In addition to these criteria, other findings are also relevant, including a good overall state of health, the absence of other clinical manifestations attributable to PCM, the presence of compact granulomas on histopathological examination of lesions, and a strongly positive intradermal reaction.

### 7.2 Virulence and Escape Mechanisms

The yeast phase is the pathogenic form of the *P. brasiliensis*. High concentrations of α-1,3-glucan and low concentrations of galactomannan in the cell wall of the yeast-like forms are correlated with *P. brasiliensis* virulence [104, 105].

In a murine model, gp43 antigenaemia led to depression of the cell-mediated immune response [106]. In addition, gp43 antigenaemia was adequately demonstrated in patients and may last a very long time, up to two years in patients with the acute/subacute form of disease [107]. These findings suggest that gp43 might have a considerable immunomodulating effect, maintaining the depression of the cell-mediated immune response and high serum antibody levels.

The immunogenicity and pathogenicity of *P. brasiliensis* samples freshly isolated from patients with PCM were assessed and compared to the severity of disease [108]. The results showed a direct correlation between severity of disease and virulence, especially in the patients at both extremities of the severity spectrum – mild and severe. By contrast, the immune response was found to be a host characteristic, with the infecting fungi playing a secondary role.

### 7.3 Interaction of *Paracoccidioides Brasiliensis* Complex with Host Cells

The respiratory epithelium represents the primary site at which contact between fungus propagules and hosts occurs. The cells involved in human *Paracoccidioides* sp infection include alveolar macrophages and alveolar epithelial cells [109]. Although epithelial cells serve as a relatively passive physical barrier to infection, they may contribute more actively to the signalling events that occur during immune responses [109]. Furthermore, epithelial and endothelial cells may serve as a reservoir for the fungus, protecting them from macrophages and other immune system cells [110]. The migration of pathogenic yeasts to endothelial cells is considered to be a prerequisite for multiple organ invasion and fungus dissemination [109, 111].

The use of mammalian cell culture techniques has provided unique insights into these host-fungus interactions. A cell line derived from human alveolar epithelial cells (A549 cells) has been used as an *in vitro* type II pulmonary epithelial cell model, as have been Vero and HeLa cells [112]. These models have been developed to study the processes that occur between initial host-*Paracoccidioides* sp contact and the events that culminate when fungal cells enter the host [113]. Fungal adhesion processes have been reported to vary between strains and correlate with virulence [114], and strains that are more virulent in animals have been found to exhibit enhanced adhesion *in vitro* [115].

The ability of pathogens to colonize their hosts is highly dependent upon the mechanisms that allow the pathogen to overcome the physical and immunological barriers imposed by the host. To avoid rapid clearance, pathogens may quickly and effectively adhere to host cells. The capacity of the type of the cells to interact with each other in an orderly manner depends on multiple adhesive interactions between cells and their adjacent extracellular environment, mediated by cell adhesion molecules [116, 117] that function as cell surface receptors, that can trigger physical and biochemical signals that regulate a great numbers of functions, such as cell proliferation, gene expression, differentiation, apoptosis and cell migration and are used as a gateway to some pathogens [118-121].

The internalization of many pathogenic microorganisms by epithelial cells may be associated to the ability of these organisms to induce this process, forcing the activation of phagocytosis mechanisms and resulting in these cells behaving like “unprofessional phagocyte”, thereby providing a mechanism by which yeast cells can evade the professionalphagocytes and potentially facilitating the dissemination ofpathogens [122]. For this process to occur, specific extracellular signals stimulate, cytoskeleton rearrangement at the site in which contact with the microorganism occurs [123, 124], involving integrins and the cytoskeleton [113, 125]. The involvement of integrins in these processes could be linked to the interaction between fungal cells and the host lipid membrane rafts and associated with the production of certain types of interleukins. Therefore, the disruption of epithelial cell membrane rafts by nystatin, for example, has been found to be associated with decreased IL-6 and IL-8 levels in *Paracoccidioides* sp/A549 cell cultures. Therefore, these interactions have been found to be associated with increases in the host’s α3 and α5 integrins levels and the clustering of receptors onto membrane rafts, suggesting that *Paracoccidioides* sp may modulate host inflammation [126].

The structures of the cytoskeletons of pulmonary epithelial cells and keratinocytes and their morphological features, including actin, tubulin and cytokeratin, could be affected by the interactions between the host and *Paracoccidioides* sp [127, 128]. Keratinocyte parasitism may represent a possible mechanism by which fungal cells can evade local immune mechanisms [129]. Furthermore, cytochalasin D and colchicine treatment have been found to reduce *Paracoccidioides* sp invasion, indicating the functional involvement of microfilaments and microtubules in this process [127, 130].

Some *Paracoccidioides* sp proteins, referred to as adhesins, may be mediating the cell invasion process. The 43 kDa glycoprotein (gp43) may also participate in cytokeratin degradation, leading the loss of the filamentous characteristics and facilitating the invasion of the host [130-134]. Additionally, the 14-3-3 adhesin has been recognized to have the capacity to cause structural modifications in host cells, thereby influencing polymerization of the cytokeratin microfilaments of actin [135-139].

Members of the Rho GTPase family of proteins have been observed to regulate the dynamic organization of the cytoskeleton and membrane traffic related to physiological processes such as cell proliferation, motility, polarity and growth [140]; thus, these proteins may play an important role in the interaction between fungal and mammalian cells. The activation of the tyrosine kinase (PTK) receptors that stimulate Rho GTPase subsequently activates the Ras pathways and MAPKs [140]; during a previous evaluation of this process, pre-treatment of epithelial cells with genistein resulted in a significant inhibition of fungal invasion, suggesting that the inhibition of PTK is important in signal transduction during early events in the epithelial cell adhesion and invasion processes of *Paracoccidioides* sp [141]. Another important family of kinases involved in the *Paracoccidioides*-host interaction is the Src family (SFKs), and the host-fungus interaction process may involve the activation these kinases and extracellular signal-regulated kinase 1/2 (ERK1/2) in the affected epithelial cells. These data indicate that epithelial cell membrane rafts are essential for the adhesion to and activation of cell signalling molecules by *Paracoccidioides* sp [142]; for example, cytokine secretion was reported to be dependent upon p38 mitogen-activated protein kinase (MAPK), c-Jun NH2-terminal kinase (JNK) and extracellular signal-regulated kinase (ERK) 1/2 activation, and the secretion of IL-8 and IL-6 that is promoted by this fungus have been reported to be dependent upon activation of p38 MAPK and ERK 1/2 in A549 cells [143].

The ability of these pathogens to induce apoptosis may be an important virulence factor since it reduces the host's defence mechanisms [144]. *Paracoccidioides* sp induce apoptosis when they invade epithelial cells or phagocytes, benefiting the intracellular survival of this fungal species [127, 145, 146]. Furthermore, the induction of macrophage apoptosis has been found to be associated with expression of caspase-2, 3 and 8 [147]. Moreover, *Paracoccidioides* sp may modulate epithelial cell (A549) apoptosis *via* the expression of apoptotic molecules, such as Bcl-2, Bak and caspase-3, supporting the hypothesis that apoptosis may be induced by the fungus to promote its survival and dissemination [148, 149]. More recently, Silva and colleagues [150] showed that the 14-3-3 and gp43 adhesins had substantial influences on this process. Additionally, apoptosis may be mediated by Fas-FasL, and CTLA-4 was identified to be involved in modulating immune responses in patients infected with PCM [151].

To further increase the understanding of *Paracoccidioides*-host interactions, the use of mammalian cells models may facilitate the establishment of increased knowledge related to how fungus-host interactions have evolved to enable the fungi to evade the immune system of the host and why *Paracoccidioides* sp are the organisms associated with the highest incidence of mycosis in Latin America [130].

### 7.4 Pathology – Histomorphologic View

The pathological findings of PCM will be focused on the pulmonary involvement. It is characterized by granulomatous inflammatory processes, during which the intensity and morphologic pattern depend on the immune status of the host, duration of infection without treatment and virulence of the fungus [152].

Typically, performing hematoxylin and eosin staining on pathological specimens from immune-competent patients reveals multiple well-formed granulomas composed of cohesive clusters of histiocytes and organized centrally with peripheral admixed lymphocytes; however, some eosinophils and/or neutrophils have been detected Fig. (**1C**) [153, 154]. These histiocytes are also characterized by broad, foaming cytoplasm called epithelioid, and giant cells are occasionally observed in their inflammatory infiltrate [153]. Both cells may engulf a relatively small number of *P. brasiliensis* forms in granulomas; however, they may also course freely within the connective tissue. Moreover, necrosis is not frequently observed but may occur in some cases.

According to the “grade” of immunity, granulomas may be extensive with dispersed macrophages, lymphocytes and plasmocytes. On the other hand, immunocompromised patients may develop pneumonic reactions with diffuse alveolitis composed predominantely of neutrophils and affecting both the hilar and peripheral regions of the lungs [153, 155].

Potential *P. brasiliensis* infections are generally evaluated based on typical histomorphological features through the use of light microscopy in appropriate clinical settings. A multinucleated cytoplasm that is separated from the cell wall with a clear space or halo may sometimes be recognized in H&E-stained sections. To better diagnosis PCM, Gomori’s methenamine silver staining (GMS) should be performed to determine the morphological aspects of this fungus. Through the use of GMS and other staining methods, such as Papanicolaou and periodic acid-Schiff (PAS) staining, the yeast cells of *P. brasiliensis*, which are spherical, vary markedly in size, and have multiple buds, or a “pilot wheel” appearance may be revealed [155].

In general, the injuries resulting from PCM are centred on the bronchovascular axes of the proximal airways and associated with radiological findings consistent with a butterfly wing pattern. In patients with chronic disease, the granulomatous inflammatory process promotes myofibroblastic activation and increased extracellular matrix deposition [155]. Consequently, parenchyma and vascular lung remodelling result in improvements in radial fibrosis, traction bronchiectasis and lung architectural distortion. The final outcome is a pulmonary fibrosis, sometimes accompanied by *cor pulmonale* and death [152].

## 8 AUTOPSY FINDINGS

Table **1** describes the prevalence of lesions in several organs as found in necropsy studies. The data indicate the predominant involvement of the lungs, lymph nodes, mucous membrane of the UADT and adrenal glands [156-163].

## 9 IMMUNE RESPONSE

The onset, progression, and clinical outcomes of PCM are influenced by environmental factors, the host’s immune responses and genetic background [164]. Compelling evidence suggests that immunity against *Paracoccidioides* sp is based on three major principles: 1) PCM is an endemic disease and affects healthy individuals, *i.e.*, those without immunosuppressive underlying conditions, such as neoplasia or use of immunosuppressive drugs; 2) the adaptive immune response against *Paracoccidioides*-specific antigens is deficient and may modulate the immune responses to other antigens [165]; and 3) host responses are dependent upon gender, nutritional status, size of inhaled inoculum, and possibly genetic background.

In *Paracoccidioides* sp, cell death may be induced by hydrogen peroxide (H_2_O_2_) produced by macrophages [166], which is enhanced by the T helper 1 (Th1)-polarized immune response [167]. Effector Th1 lymphocytes are recruited to the site of infection and release IFN-y, which enhances macrophage activation. Therefore, disturbances in the orchestration of these mechanisms may lead to the onset and progression of disease.

The duration of the symptomatology of PCM is short in patients with the AF, ranging from a few weeks to some months (median of 2 months) [168], and prolonged in patients with the CF, usually higher than 6 months [169]. Independent of the clinical form of PCM, hosts initiate an adaptive immune response and, by the time of patient admission, this response has already become polarized. Although the initial events of the *Paracoccidioides*-host interaction have been well explored using experimental models, in the present review, we mainly focused on the immune features observed in PCM patients.

PCM patients show a wide spectrum of clinical manifestations, which are correlated with the type of immune response activated [170, 171]. The adaptive immune response involves highly specific interactions between immune cells and soluble factors such as antibodies, cytokines and fungal antigens. In general, the clinical forms of PCM exhibit dichotomous Th responses: whereas patients with the AF of the disease have abundant antibodies but poor to nil T cell/cell mediated immune responses, patients with the CF of the disease demonstrate good T cell responses, as indicated by skin tests and **in vitro** correlates of T cell immunity [170, 171]. An overview of the polarization of adaptive immune responses in PCM patients is shown in Fig. (**4**).

The Th2 / Th9 type of immune response is characteristic of the acute clinical form of PCM (Table **2**). Patients with the AF of the disease exhibit high IL-4, IL-5 and IL-9 levels and nonreactive paracoccidioidin skin tests. This finding reflects the marked depression of cellular-mediated immunity that occurs in these patients. Furthermore, these patients produce large amounts of antigen-specific IgA, IgE and IgG4 [172], an antibody isotype that has been found to exhibit diminished complement fixation capacity and a low affinity for the FcR receptors present in phagocytes, resulting in poor phagocytes is by macrophages and subsequent fungal multiplication and dissemination.

In patients with the CF of the disease, following disruption of the prolonged fungus-host equilibrium, the reactivation of latent foci (endogenous reinfection) leads to disease progression. Regardless, the Th1 response is more preserved in these patients (Table **2**) since the majority of them has reactive paracoccidioidin skin tests, except for those with severe forms of the disease. In addition, these patients exhibit increased production of pro-inflammatory cytokines, such as TNF-α, IL-1β, and IL-17, and hydrogen peroxide (Table **2**). Although these mediators are important for fungal elimination, their production reflects the host's inability to limit the *Paracoccidioides* infection, as the lysis capacity of the host’s immune system cannot restrict the spread of the fungus. Furthermore, these cytokines may have deleterious effects on patients, such as anorexia, cachexia, and cell death [173]. The levels of antibodies may also be high; however, this response is characterized by IgG1 and IgG2 isotype antibodies [172], which show high complement fixation capacity and high affinity for FcR receptors (IgG1> IgG2> IgG4).

Active regulatory immune responses are present in both clinical forms of PCM and have been characterized by the expression of FoxP3 in tissue lesions and high production of IL-10 and TGF-β1 by peripheral blood mononuclear cells (Table **2**). Regulatory T (Treg) cells act by counterbalancing immune responses during persistent infections to promote the control of immune-mediated pathology while avoiding overactive inflammatory responses. On the other hand, several studies have noted the detrimental role that host defences play in preventing microbial elimination [174].

In both clinical forms, host immune responses influence the severity of the disease. In critically ill patients, regardless of the clinical form of the disease, the impairment of cellular immunity is more pronounced. Thus, these patients present tissue lesions typically characterized by extensive granulomas, foamy macrophages, high amounts of fungi, few peripheral lymphocytes and increased Th2 cytokine production, as indicated by immune staining methods [168, 171]. In addition, these patients show high titres of specific antibodies, and anergy at paracoccidioidin skin tests [29]. On the other hand, in patients with moderate and mild forms of the disease and in whom the immune response is more preserved, the histopathological features of PCM are typically characterized by well-organized granulomas composed of epithelioid cells, giant cells, a few live yeast cells, dead fungal biomass, and a thickened halo of peripheral lymphocytes [175]. Additionally, lower specific antibody titres and positive reactions to the paracoccidioidin skin test are observed in these patients [29].

Although seldom studied, these immune responses may be modified during antifungal treatment. The titres of circulating antibodies diminish as cellular immunity is recovered over the course of treatment [170]. This recovery is a slow process that depends on a reduction in antigenic load resulting from effective antifungal therapy, among other factors. The recovery of cellular immunity is essential to prevent relapses caused by the proliferation of quiescent yeasts after removal of the antifungal agent [176, 177]. In addition, patients with the CF of the disease have been found to show persistent nonspecific inflammatory responses during and even after successful antifungal therapy, which are characterized by increased production of TNF-α [178], activation of the NLRP3 inflammasome, and high counts of CD14^+^CD16^++^ inflammatory monocytes [178]. The immunological alterations observed in patients with the CF of the disease during and after treatment may be associated with hypoxia due to pulmonary fibrosis and emphysema. Activation of some transcription factors, such as hypoxia-inducible factors (HIF) [179], may induce growth factor signaling, proinflammatory cytokine release, co-stimulatory molecule expression and lymphocyte proliferation [180, 181].

Typically, patients with the CF of the disease present with fibrosis at their initial admission [182] and show enhanced production of TGF-β1 and the basic fibroblast growth factor (FGF-b), as shown in Table **2**. Previous autopsy findings for PCM patients have demonstrated that pulmonary fibrosis is characterized by extensive areas of collagen deposition in the hilar region and the involvement of other structures such as the lymph nodes, bronchi and arteries. These collagen fibersfrequently border granulomas and extend to the bronchi and nearby blood vessels. The proliferation of reticular fibers (collagen III) in the alveolar septa also occurs, including fibrotic areas of the granulomas [153]. Though many challenges remain to be overcome regarding PCM fibrogenesis, accumulating evidence suggests that fibrosis occurs as a result of the prolonged inflammation, constant antigen stimulation and persistent parenchymal injury that induce the natural wound healing process [183]. Chronic inflammation may also induce tissue damage and parenchymal cell death due to necrosis or apoptosis, and local production of cytokines and chemokines activate neighbouring cells to produce pro inflammatory cytokines and pro-fibrogenic growth factors such as TGF-β1 and FGF-b. These mediators stimulate the production of collagen by fibroblasts, establishing fibrosis. An overview of PCM fibrogenesis is proposed in Fig. (**5**).

## 10 CLINICAL MANIFESTATIONS

As a systemic mycosis with remarkable tendency to spread and affect any organ or system, PCM exhibits polymorphous clinical manifestations. For this reason, PCM infection is often confounded with other diseases, especially among females and younger patients.

In general, patient complaints include feeling unwell, anorexia and weight loss, which might be so severe as to cause cachexia. Fever is occasionally present and should be considered a sign of greater severity. The clinical manifestations associated with the involvement of various organs are described below, followed by the classification of the clinical forms of disease.

### 10.1 Involvement of Organs and Systems

#### 10.1.1 Lungs

Lung involvement is particularly relevant due to its high frequency and occurrence of residual fibrosis as well as because the lungs are the portal of entry for *P. brasiliensis* in almost all of the patients.

The first case of pulmonary involvement by PCM was reported in 1911 [190], and the first case with exclusively affected lungs, without clinical manifestations compatible with extra-pulmonary lesions, was published eight years later [191]. The relevance of lung participation was recognised only in 1946, when it was detected in 84% of 25 autopsied cases [15].

An assessment of patients with PCM who were non-smokers and did not exhibit any other respiratory disease reported cough in only 57% of the cases and expectoration in 50%. The sputum was almost always mucous, but bloody in some cases (11%). In general, the patients did not report chest pain [192]. Dyspnoea, the most frequent complaint, first appeared on heavy exertion and had a progressive character, manifesting even at rest. Lung involvement, however, can also be asymptomatic.

Physical examination of the lungs does not yield many signs, even among patients with severe respiratory symptoms, characterising a clinico-semiological dissociation. The respiratory examination may be normal in up to 43% of patients with PCM lung lesions [192].

Plain chest radiographs primarily indicate interstitial or mixed (alveolar-interstitial) lesions, with a predominance of interstitial abnormalities. These lesions are usually bilateral, parahilar and symmetrical, most often located on the middle third of the lungs. The upper third is affected in approximately one-third of cases and the apex in half, bilaterally. Among the interstitial lesions, the reticulonodular ones predominate [193].

Alveolar or mixed lesions, with predominance of the former, are also bilateral, parahilar and symmetrical, usually sparing the lung apex and base. The overall aspect is evocative of butterfly wings, an image highly suggestive of *Paracoccidioides* infection, although its prevalence is low Fig. (**6**).

In addition to the aforementioned patterns, the radiological abnormalities might bear resemblance to a tumour, pneumonia or a cavitated mass [154, 193]. Occasionally, the radiological findings mimic those observed in tuberculosis.

Lung cavities were first described by Fialho [15], being characterised as irregular excavations of up to 2.0cm in diameter and containing a viscous exudate. The pressure exerted by neighbouring tissues narrows the cavities down to tortuous slits. In association with severe parenchymal involvement, this morphology makes their visualisation on plain chest radiographs difficult. However, these abnormalities are well identified on conventional chest tomography (planigraphy), where they appear as multiple rounded lesions, usually being smaller than 2.0cm at the largest diameter and exhibiting thick walls. Some of the cavitated lesions may be confluent [194].

Involvement of the hilar and mediastinal lymph nodes was also detected on autopsy [15], findings which are seldom detected on plain chest radiographs. The severe compromising of the parenchyma, which is most evident close to the hila, impairs the observation of hilar structures. However, in 50% of the cases, chest planigraphy is able to reveal lymph node enlargement [194].

Computerised axial tomography of the chest represented a major contribution to the understanding of PCM lung lesions. In untreated patients, nodules predominate, especially small nodules. Other findings include septum thickening, thick lines, alveolar opacities, blocks of fibrosis, bronchial wall thickening, bronchiectasis, and cavities without fluid content [195]. Shortly after the onset of treatment the frequency of bronchiectasis, bullae and diffuse emphysema tends to increase [195].

On high-resolution computerized tomography (HRCT), thickening of the interlobular septa is the most frequent finding (92% of cases) but is sparse and low intensity. Thickening is followed in frequency by a)areas of emphysema (69%); b) areas of ground-glass attenuation (62%); c) bronchial wall thickening (54%); d) tracheal dilation (46%); e) nodules (39%), cavities, architectural distortion, spiculated pleural thickening and parenchymatous bands (31%); and f) areas of consolidation, intralobular reticular thickening and axial interstitium thickening with bronchovascular distortion (23%) [196].

A clinico-radiological dissociation, characterized by a low frequency of respiratory complaints among patients with pulmonary involvement, sometimes extensive, on radiological examination, has been a frequent finding [8, 12, 16, 103].

Pulmonary involvement is rare among young patients; however, it may occur in 5 to 11% of cases [26, 197-199]. For this reason, diagnosis is confirmed only on autopsy. Thus, in endemic areas, this possibility should be taken into consideration whenever a patient exhibits epidemiological antecedents for PCM or the clinical progression is not satisfactory after the use of antimicrobial therapy for common lung disorders.

Pulmonary function is usually abnormal, with the obstructive pattern being the most frequent, followed by a mixed pattern; very few patients exhibit a restrictive pattern [200]. Hypoxaemia occurs in almost all patients, and the alveolar-arterial oxygen gradient is increased in practically all cases, reflecting a predominance of perfusion over ventilation. Some data suggest that the air and blood distribution, as well as diffusion, might be altered in the very early stage of the disease. Patients who exhibit an obstructive pattern present with early airway involvement as well as changes in the ventilation/perfusion ratio, alveolar diffusion and ventilation. These abnormalities were also detected in patients with a mixed pattern, showing that obstructive lung disorders predominate in PCM. The findings on spirometry suggest that bronchial involvement predominates in PCM, especially at the level of the bronchioles or of the peribronchiolar connective tissue, both in the early and late stage of disease; these changes have no relationship to smoking [191]. These suggestions are based on a careful autopsy study that evidenced granulomas and areas of fibrosis surrounding the bronchi, which were attached to other bronchi and blood vessels *via* fibrous septa [160].

The regression of radiological lesions after the onset of treatment is not attended by recovery of pulmonary function [201]. As the proliferation of collagen and reticulin fibers is not always associated with the occurrence of granulomatous reaction but with the presence of *P. brasiliensis*, it is suggested that the fungus *per se* triggers reticulin proliferation [160].

The initial respiratory symptoms decrease or fully disappear after the onset of treatment. In general, patients complain of persistent morning cough, attended or not by hyaline expectoration. Many patients initially exhibit dyspnoea on heavy exertion, which might become worse, appearing with moderate or even mild exertion. Plain chest radiographs reveal lung sequelae characterised by fibrosis, diffuse or bullous emphysema, and occasionally pulmonary hypertension. Chest computerized tomography shows alveolar opacities (24% of cases), nodules (38%, mainly small), septal thickening (100%), bronchial wall thickening (89%, usually mild), bronchiectasis (41%, usually mild), bullae (59%), diffuse emphysema (70%) and pleural thickness (65%). Evidence of cavities and “honeycomb” lesions are infrequent. Patients usually do not exhibit hilar or mediastinal lymph node enlargement [182]. The pulmonary function is seldom normal; 85% of patients exhibit the obstructive pattern, and the frequencies of mild, moderate and severe degrees of obstruction are equal. Hypoxaemia occurs in approximately one-third of cases as a sequel [182].

##### 10.1.1.1 Pleura

Pleural involvement is detected in only 2% of cases on plain chest radiographs and is characterised by small effusions and thickening [193, 202].

Pleural involvement is quite rare, as pleural effusion [202] or spontaneous pneumothorax [203].

Pleural effusion in PCM was observed predominantly in patients with the chronic form, most of them presenting no comorbidity [202]. The pleural effusion can be caused by alterations of the permeability due to its paracoccidioidal involvement arisen from a parenchymal process. Another possibility is the inflammatory injury to the visceral pleura microcirculation that disrupts the pleurolymphatic drainage. It is worth noting the finding that 60% of the autopsied patients presented thickening of pleura without an effusion [15]. The diagnosis of the paracoccidioidal pleural involvement depends on the identification of the aetiological agent in tissue fragments and/or pleural fluid.

As cigarette smoking is a risk factor for PCM and CPOD, and CPOD can cause, by itself, secondary spontaneous pneumothorax, it is difficult to attribute this pleural disease only to PCM. However, the odds of pneumothorax in PCM patients is 4.3 times higher than in cases of CPOD [203]. The rupture of the subpleural emphysematous bullae or necrotic cavities, caused by a sudden increase in airway pressure, can be caused by caugh, allowing for communication between the pulmonary airways and the pleural space. The spontaneous pneumothorax has been observed in patients with active non-treated PCM, in cases of relapse and in patients with apparent cure [203]. Sudden onset of dyspnoea, chest pain and decreased or absent breath sounds on lung auscultation are the most important clinical manifestations of pneumothorax.

##### 10.1.1.2 Dermatological Aspects - Correlations Between Clinical and Histopathological Findings in Paracoccidioidomycosis

In patients with systemic mycosis, such as PCM, the presence of cutaneous and mucosal lesions are initiators of specific diagnoses. Taken together, the characteristics of these lesions, including their number, clinical morphology and accessibility for biopsy and collection of material for direct examination and culture, provide valuable indicators that may enable early diagnosis. In a case series including 152 consecutively enrolled patients with PCM who were followed-up at a dermatologic university service, the presence of specific cutaneous lesions was observed in 61.2% of patients, and the presence of specific mucosal lesions was observed in 58.5% of patients [204]. Overall, 90.8% of patients included in the afore mentioned study presented with cutaneous and/or mucosal lesions accompanied by lung or other systemic involvement. These numbers highlight the importance of these lesions as valuable indicators for the establishment of diagnoses.

Cutaneous lesions generally occur as a consequence of haematogenous fungal dissemination, usually originating from the lungs or contiguously evolving from mucosal lesions and often presenting with lip involvement. Remarkably, these lesions arise from direct inoculation of the dermal tissues with *Paracoccidioides* sp. The most common topographic locations of cutaneous lesions are the head and neck (47.6%), inferior limbs (21.8%), trunk and superior limbs (14.9%) and genitals (0.7%) [204]. Cutaneous lesions most frequently occur in patients with the chronic (adult) form of the disease but may be identified in patients with the acute/subacute (juvenile) form of the disease. Mucosal lesions are mainly observed on the buccal mucosa and among patient with chronic PCM. Lesions on the buccal mucosa are most commonly identified on the gingiva, soft palate, lip and jugal mucosa [204, 205]. Lesions occurring on the tongue or tonsils are less commonly observed.

The clinical features of cutaneous lesions occur as a result of tissue responses to the presence of fungal cells in the dermal tissues. This host-parasite interaction is a dynamic process, and modification of the immune response may result in the formation of different types of clinical lesions over time in the same patient or differences in the number and clinical features of lesions among diverse patients. Ulcers are the most prevalent type of lesion and may arise from pre-existing solid lesions, such as papular, nodular or verrucous lesions, or as a consequence of inflammatory events that occur in response to the presence of the fungal cells in the dermal tissues. Histologically, PCM is characterized by granulomatous inflammatory responses that occur around one or more fungal cells. Pedagogically, the existence of two polarized clinical expressions of the disease, one “hyperergic” and the other “anergic”, could be conceived, a concept first proposed by Lacaz in 1982 and more thoroughly explored by Del Negro *et al.* in 1994 [206]. Between these polarized forms, many possible clinical expressions may occur based on host-parasite interactions. On the “hyperergic” side of the spectrum, we would expect patients to have fewer lesions and, possibly, sarcoid reactions, with granulomas that are compact and made up of giant and epithelioid cells. In these patients, the INF-γ and IL-12 (Th1) immune responses [207] and cells expressing the Foxp3 and CD25 markers may predominate [208]. In these cases, the inflammatory response is rich in lymphocytes, which surround the epithelioid cells, and poor in polymorphonuclear leukocytes, and the fungal cells are few and contained by inflammatory responses Figs. (**7A**, **B**). When affected by the anergic form of the disease, patients often present with poor clinical and nutritional status and a large number of cutaneous lesions that are initially acne form but then evolve into ulcerative and necrotic lesions. In these cases, IL-5 and IL-10 production and the Th2 immune response prevail [207], and subjacent causes of immunosuppression should be investigated [209]. The granulomas affecting these patients are poorly organized, oedematous, rich in polymorphonuclear leucocytes, and usually have central suppuration and coagulation necrosis. The number of fungal cells in these patients is high, with many cells multiplying and many cells of minute size Figs. (**7C**, **8A**).

Verrucous lesions are generally observed in patients with more balanced host-parasite equilibrium, a low to moderate number of cutaneous lesions, and good clinical condition. Verrucous lesions correspond to a type of tissue response characterized by marked pseudoepitheliomatous hyperplasia Fig. (**8C**). This type of lesion requires clinical and histological differentiation from squamous cell carcinomas. The presence of inflammatory granulomatous infiltration accompanied by fungal cells in the dermal tissues may help makes this distinction clear; however, the risk of misdiagnosis increases when superficial or shave biopsies are performed.

Mucosal lesions in PCM patients are characterized by superficial ulcers with microgranulation and haemorrhagic pinpoints, often referred to as mulberry-like stomatitis. These mucosal lesions also exhibit infiltrated borders or infiltrative tissue at their base. This clinical feature is often observed in patients with lesions of the buccal, ocular or genital mucosa. The histological features of mucosal lesions are similar to those of ulcerated cutaneous lesions.

Methenamine silver nitrate (Grocott-Gomori stain) and periodic-acid of Schiff (PAS) stains are frequently used to better detect the presence of *Paracoccidioides* in dermal tissues Fig. (**8B**).

##### 10.1.1.3 Lymph Nodes

Involvement of the (submandibular) lymph nodes was first reported by Lutz [1]. *P. brasiliensis* lymphotropism was suggested by Haberfeld (1919) [210], and Niño (1939) pointed to a direct relationship between poor prognosis and the early appearance and severity of lymphadenopathy [211].

The relevance of lymph node involvement may be assessed based on its frequency in clinical and autopsy studies, the detection of subclinical involvement, the alterations of the lymphatic system identified by lymphographic and scintigraphic evaluations, and more particularly the depression of the cell-mediated immune response resulting from lymphoid tissue damage.

*Paracoccidioides* sp may spread to the lymph nodes *via* the haematogenous or lymphatic routes. The fungus is drained from organ lesions to the regional lymph nodes and then spreads to other lymph nodes *via* the lymphatic system. The haematogenous route allows the fungus to spread to lymph nodes through the arteries that feed them.

Subclinical lymphadenopathy, defined by the detection of paracoccidioidallesions in lymph nodes that are considered normal on clinical examination, was found in the lymph nodes that drain affected areas as well as in others quite distant from fungal lesions [212-214]. The latter situation is admittedly caused by haematogenous spread.

Lymph node enlargement may be the main clinical complaint, being the rule among children, adolescents and young adults, who exhibit the acute/subacute form of PCM, also known as “juvenile form” [26, 195, 198, 199, 215-217].

The lymphatic chains most often affected are those of the head, followed by the supraclavicular and axillary nodes [212]. In the head, the submandibular and anterior and posterior cervical nodes are most frequently involved. The submental, tonsillar, pre- and post-auricular, and even the suboccipital lymph nodes may also be affected with variable frequency. Although rare, intercostal, epitrochlear and popliteal lymph node involvement was described, primarily in severe cases.

Abdominal lymphadenopathy, originally described in 1915 [218, 219], has been frequently reported in the Centre-West region of Brazil as well as in the Botucatu area [220, 221], occasionally with clinical manifestations that mimic acute abdomen [219, 222]. The presence of large tumour-like masses on palpation is suggestive of lymphoproliferative disease. Abdominal lymph node enlargement can cause extrinsic compression. Obstructive jaundice is not uncommon among patients with involvement of the hepatic hilum lymph nodes and compression of the extrahepatic bile ducts [223, 224]. In addition, inferior vena cava syndrome was described in a patient with PCM and abdominal lymphadenopathy [225].

Mesenteric lymphadenopathy can lead to malabsorption, occasionally attended by chylous ascites [226-228]. Involvement of the deep lymphatic system, the location of which makes difficult its characterisation on physical examination, can be assessed by ultrasound [229], computerised axial tomography (CAT) [230], lymphography [229, 231-234] or lymphoscintigraphy [235].

Patients with the chronic form of PCM may exhibit cervical and submandibular lymphadenopathy in relation with the drainage of lesions of the mucous membrane of the UADT. However, it is worth noting that patients with the PCM chronic form and without lymphadenopathy on physical examination were found to exhibit severe involvement of the deep lymphatic system on bipedal lymphography [234].

The clinical characterisation of lymphatic involvement is overall difficult because different lymph nodes from various chains may be affected and exhibit different characteristics in the same patient. For this reason, lymphatic involvement is clinically classified into three types based on the largest diameter of the lymph nodes and the presence, or not, of suppuration, as follows: 1) inflammatory non-suppurative: the largest diameter of all lymph nodes is less than 2.0cm and none exhibits suppuration; 2) tumoural: no lymph node exhibits suppuration and the diameter of at least one is ≥2.0cm; and c) suppurative: at least one lymph node exhibits fluctuation or fistula, independently from its diameter Fig. (**9A**). The lymph nodes of patients with the inflammatory non-suppurative type tend to be painless, non-coalescent and mobile, without exhibiting either heat or redness. In patients with tumoural lymphadenopathy, the lymph nodes are usually painful on palpation, fixed to deep or superficial planes and coalescent, with redness and/or heat [236]. It is worth noting that when classifying the type of lymphadenopathy, the established type applies only to the time of assessment; if patients do not receive appropriate treatment, or at times in spite of it, the infection may progress, and the lymph nodes may become even larger and/or suppurate.

Bipedal lymphography affords an excellent morphological assessment of the lymphatic system. The lymphangiographic phase shows dilation, segmentation, delay in outflow, and, less often, obstruction of the lymphatic vessels. The lymphographic phase reveals abnormalities in filling, shape, size, form of presentation, and number of opacified lymph nodes [234]. Patients with the chronic form of PCM exhibit symmetrical lymphographic alterations [234].

Lymphoscintigraphy affords an excellent functional assessment of the lymphatic system. This test allows the investigation of the lymphatic flow by means of semi-quantitative and quantitative variables and lymph node uptake of the radiotracer [235]. In patients with the acute/subacute form of PCM, the lymphatic flow is increased in the lower limbs before antifungal treatment. The low serum albumin levels exhibited by this population of patients might account for this finding. In this population, the flow pattern does not change after the first few months of treatment; in contrast, patients with the chronic form of PCM exhibit increased lymphatic flow after the onset of treatment [235].

### 10.2 Otolaryngological Aspects

The involvement of the mucous membrane of the upper aerodigestive tract - UADT, *i.e.*, the nasal fossa, oral cavity, oropharynx, hypopharynx and larynx, is highly relevant due to its high frequency and the ease of collecting samples for the identification of the aetiological agent [237-241].

Aguiar Pupo (1936) conducted the first systematic study of lesions in mucous membranes caused by *P. brasiliensis* and described moriform ulcerative stomatitis, which later on was given his name [237].

Hoarseness, pain and difficulty swallowing, burning in the throat, the feeling of bulging or a wound in the mouth and dyspnoea are the most common clinical manifestations [241]. The lesions in the mucous membrane might be very painful, especially when eating hot or very salty foods. In general, more than one UADT area is involved, with most lesions being in the larynx, followed by the oropharynx, hypopharynx and oral cavity, which exhibit similar prevalences. Bilaterality is almost always observed for all sites, but the morphology of the lesions is highly variable. Hyperaemia, moriform lesions, swelling, granulomatous infiltrative lesions, ulcerations, granular lesions, infiltrative lesions, and vegetating lesions have all been described. The progression of Aguiar Pupo’s moriform ulcerative stomatitis is slow, and its presence is suggestive of PCM. This form is characterised by an ulcerated area, the base of which has a fine granular, mulberry-like appearance. Together with hyperaemia, it is the type of lesion that predominates in the oral cavity. In the oropharynx lesions affect more frequently the soft palate and anterior and posterior pillars, followed by the lateral and posterior walls, uvula, tongue, and tonsil area. Hypopharyngeal lesions are distributeduniformly among the lateral, anterior and posterior walls and the pyriform sinus, and hyperaemia and moriform lesions are the predominant lesion types. All the areas of the larynx may be affected, more frequently the ventricular fold, arytenoid area, vocal cords and the free portion of the epiglottis, followed by the laryngeal surface of the epiglottis, aryepiglottic fold, subglottic area and the laryngeal-ventricle space. Swelling, granular lesions, hyperaemia and moriform lesion are the most common lesion types, having similar prevalences. Vegetating lesions and ulcers are found in very few cases. Involvement of the nasal mucous membrane, columella and nasal septum has also been reported.

The gums are frequently involved, accompanied by teeth loosening. These findings are suggestive of PCM, make eating difficult and havea negative impact on the patient’s nutritional status [238, 241].

Lastly, hard palate perforation must be mentioned, although it is a rare occurrence [242].

The functional study of laryngeal sequelae in PCM patients revealed frequent and severe dysphonia (dysphonia index <7.0), characterized by roughness and breathness. The Dr. Speech (Tiger Electronics) analysis program did not accept five out of 15 voices due to severe dysphonia. Jitter was elevated in five patients [243]. The severe dysphonia observed in patients with laryngeal sequelae may have important social consequences for PCM patients.

### 10.3 Adrenal Glands

Adrenal involvement by *P. brasiliensis* was first reported by Viana (1913 [244], 1914 [245]) during the autopsy of a patient with disseminated disease; it was subsequently reported in a patient with areas of pulmonary fibrosis [246].

It was in 1952 that the signs and symptoms exhibited by PCM patients were correlated with clinical manifestations of chronic adrenal insufficiency [247]. The Thorn test, for assessment of the adrenal function, was applied to PCM nine years later [248]. This test revealed a high incidence of adrenal involvement, which thus came to be considered as the third most frequent location of disease, and that the adrenal reserve was reduced in 48% of the assessed patients [248].

The tropism of *P. brasiliensis* for the adrenal glands might be explained by the local reduction of the cell-mediated immunity caused by its high glucocorticoid concentrations [249].

The main signs and symptoms of chronic adrenal insufficiency in patients with PCM are malaise, fatigue, anorexia, weight loss, arterial hypotension, orthostatic hypotension, hyperpigmentation of the skin and mucous membranes, nausea, vomiting, and reduced libido and sexual potency. Hyperpigmentation is usually reported or confirmed by patients and is most evident on the oral mucosa, nipples, penis, areas exposed to friction, such as the elbows, and scars. The serum potassium, calcium and urea levels are usually elevated and the sodium and chloride levels decreased.

The diagnosis of adrenal insufficiency is established based on urinary 17-hydroxycorticosteroid levels and plasma cortisol levels before and after adrenal stimulation with semisynthetic adrenocorticotropic hormone (ACTH). The baseline levels are low, and the response to stimulation is insufficient or absent [248, 250].

Evaluation of serum aldosterone levels before and after adrenal stimulation with semi-synthetic ACTH showed low levels before stimulation in several patients, and an absence of response to ACTH in some cases [250].

The plasma ACTH levels are high in patients with PCM and clinical manifestations compatible with Addison’s disease [251], suggesting that the measurement of ACTH might be useful for early diagnosis of chronic adrenal insufficiency.

Diagnostic imaging is a significant contribution to the identification of adrenal involvement in PCM. On computerized tomography, the adrenals exhibit irregular contours, as well as volume and density abnormalities. Ultrasound allows for assessing the shape, contours, density and size of the adrenal glands.

Comparison of findings on computerized tomography and ultrasound with the plasma cortisol and aldosterone levels before and after stimulation with ACTH revealed limited adrenal reserve in 53% of patients, abnormalities on computerized tomography in 43%, and ultrasound abnormalities in 17% [251]. The combination of both imaging methods allowed establishing a diagnosis of adrenal involvement in 85% of cases [251].

The adrenal function seldom recovers after antifungal treatment for PCM, but persistence of residual adrenal insufficiency is much more frequent [252].

### 10.4 Digestive System

Involvement of the gastrointestinal tract was reported in the earliest studies of patients with PCM. One of the cases reported by Lutz exhibited chronic diarrhoea, the aetiology of which was not established [1]. Viana described a case of disseminated PCM, and the autopsy revealed mycotic ulcerations in the ileum, appendix and colon. The introduction of new techniques for the investigation of the gastrointestinal tract led to more reports of new cases, although studies with large case series remain scarce, and studies assessing the full extension of the gastrointestinal tract are even rarer [253-256].

Gastrointestinal complaints are reported by more than 50% of patients when interrogation targets the digestive system. In such cases, sialorrhoea, dysphagia, halitosis, abdominal pain, bloated feeling, pyrosis, and abnormalities in intestinal motility are frequent findings, followed by regurgitation, vomiting, hiccups, and presence of an abdominal mass [256].

In another study, the most frequent clinical manifestations were abdominal pain, changes in bowel habits, nausea, and vomiting [255]. Abdominal pain is usually of the colic type alone or alternating with continuous pain. Diarrhoea lasts more than 15 days and usually consists of two to six liquid or pasty stools per day. Some patients report the presence of blood streaks or mucus in the stools. Constipation is as frequent as diarrhoea, lasting up to 10 days and usually being due to extrinsic compression or isolated intestinal lesions. Constipation may also be associated with severe obstructive disorders; for this reason, patients require careful follow up [255]. Some patients undergo periods of alternating constipation and diarrhoea. It should be noted that gastrointestinal symptoms occur more frequently among patients with the acute/subacute form of the disease and clinical evidence of involvement of the abdominal lymphatic system, which is characterised by palpable masses [255]. Gastrointestinal manifestations may be the earliest complaints among such patients.

Radiological investigation of the gastrointestinal tract reveals anatomical or functional abnormalities in 89% of patients. In most cases, such abnormalities involve more than one segment, being most frequent in the ileum, stomach, duodenum, jejunum and ascending and descending colon. Involvement of the oesophagus and rectum is rare, and involvement of the appendix is extremely rare [256].

Functional disorders are more common than the anatomical disorders. Among the former, hypersecretion, hypotonia, reduced peristalsis and barium column flocculation are the most frequent. The anatomical abnormalities with the highest incidence include mucosal fold thickening, dilation, extrinsic compression by the liver, spleen or lymph nodes, stenosis, and stiffness [256]. Some patients progress into intestinal obstruction or sub-obstruction, and consequently into acute abdomen that requires surgical treatment.

Some patients exhibit protein-losing enteropathy [227, 257, 258] and deficient absorption of glucose [226, 227, 257] and more commonly of fat [226, 227, 255, 257]. Although obstruction of the lymphatic vessels is the essential cause of these phenomena, their clinical manifestations in the gastrointestinal tract justify their inclusion in this section. These disorders are common among patients with major involvement of the abdominal lymphatic system.

Diarrhoea, chylous ascites, hypoalbuminaemia and lymphocytopaenia are characteristic of protein-losing enteropathy. Lymphatic stasis, due to abdominal lymphadenopathy, might cause hypertension of the lymphatic system, with consequent extravasation of the protein- and lymphocyte-rich lymph into the intestinal lumen. The mucosal ulcerations caused by *P. brasiliensis* may contribute to the protein loss. The presence of diarrhoea is not necessary for protein loss to occur, a factor that should always be considered whenever a patient exhibits considerable reduction of serum albumin in the absence of renal loss or impaired synthesis. Neither clinical nor radiological assessment is able to demonstrate the occurrence of protein-losing enteropathy. For this reason, the faecal excretion of ^51^Cr-labelled albumin should be investigated.

Some patients exhibit extremely fetid stools, diarrhoea, steatorrhoea, chylous ascites, and abnormal results in the plasma turbidity test after fat overload; these findings are characterised by flattened curves and increased faecal fat. Such patients often exhibit radiological intestinal abnormalities, primarily in the ileocaecal region.

Carbohydrate absorption, which is independent from the lymphatic system and takes place at more proximal regions of the small intestine, is less frequently and less severely affected compared to fat absorption and protein loss. The investigation of D-xylose absorption allows the establishment of a diagnosis of carbohydrate malabsorption.

Few studies have investigated liver involvement in PCM [223, 259-261]. The presence of liver enlargement that decreases under antifungal treatment points to PCM, especially when the tropism of *P. brasiliensis* for the mononuclear phagocyte system is taken into consideration. In general, liver lesions are not associated with clinical manifestations, although one case with intense jaundice, signs and symptoms of severe liver failure and terminal coma was reported [260]. Some patients exhibit jaundice, which is due to extrinsic compression of the bile ducts by enlarged hepatic hilum lymph nodes [223, 259].

Liver biopsy may reveal lesions with variable intensity, from mild and unspecific to severe, consisting of portal and intrasinusoidal granulomas. No patient analysed in this study exhibited signs of portal hypertension [261].

A recent study that assessed the liver and bile ducts using radioisotopes found intrahepatic cholestasis, bile duct obstruction, single or multiple focal defects and heterogeneous hepatic uptake. Intrahepatic cholestasis was most frequent among patients with the acute/subacute form of PCM [262].

Pancreatic PCM, which may mimic neoplasms of the pancreatic head or abdominal tumours, has been reported, despite being rare [263-266]. One patient had a history of severe weight loss, weakness, dizziness, general malaise, bloating, and intense itch, attended by jaundice, choluria and faecal acholia. The material obtained through computerized tomography-guided puncture aspiration of the pancreatic head contained pancreatic epithelial cells and several *P. brasiliensis* yeast-like cells [266].

### 10.5 Bones and Joints

Knowledge of the involvement of bones and joints in PCM was obtained in case reports or small case series; few prospective and systematic studies have been conducted [267, 268]. Such studies have reported a frequency of involvement between 16 and 20%. A review of studies on bone involvement published until 1964 is also available [269].

In general, *P. brasiliensis* spreads to bones via the haematogenous route, which explains its presence in patients with disseminated disease [268]. The isolation of fungi from blood cultures supports this view [269]. In addition, analysis of some cases suggests that bone involvement might also occur from lesions in adjacent tissues.

In turn, joint affection might arise from pre-existing lesions in one or more of the bones that compose the joint [270-271]. However, personal observation of a patient with joint involvement but without radiological evidence of bone affection suggests that haematogenous or lymphatic spread is a possibility.

Bone lesions begin in the medullary layer, extending first to the cortex and then to the periosteum [272]. The lesions are usually asymptomatic, and when they affect superficial bones, they might be visible or palpable; in all other cases, imaging methods are necessary to detect them. By contrast, joint affection has exuberant clinical manifestations – pain and functional impotence – with increased volume and temperature on physical examination.

Although any bone might be affected, lesions predominate in the chest (ribs and sternum), shoulder girdle (clavicles and scapulae) and upper limbs [257-269]. These locations, easily visualised on chest radiographs, are very helpful for the differential diagnosis between lung-involved PCM and tuberculosis. The presence of bone lesions in the chest, shoulder girdle or upper limbs reinforces a diagnostic hypothesis of PCM.

In general, radiographs show lytic lesions, without perifocal reaction, mild or no periosteal reaction and well-defined margins. The cortical bone is destroyed in almost half of cases. Joint involvement is detected in about one-third of cases with bone involvement [267].

Skeletal scintigraphy with 99mTc-methylene diphosphonate (MDP) is a highly valuable technique for the detection of PCM lesions due to its high sensitivity, the early appearance of abnormalities, assessment of the full skeleton in a single exam, rare contraindications, non-invasiveness and return to normal conditions after treatment [273].

The treatment of PCM is attended by fibrosis and bone neoformation, with consequent changes in the characteristics of lesions; progression is very gradual.

### 10.6 Bone Marrow

Bone marrow involvement usually occurs in patients with the acute/subacute form of PCM, being rare in cases with the chronic form. Bone marrow biopsy provides the best samples to demonstrate the host-fungus interaction [274, 275]. The lesions are variable, from focal and compact to diffuse and loose. Reticulin fibrosis predominates in the most localised lesions, while coagulative fibrosis prevails and reticulin fibrosis is discrete in more extensive and looser lesions. Residual haematopoiesis is impaired in the cases with more extensive lesions. Bone marrow involvement may contribute to the occurrence of anaemia, leukopaenia and thrombocytopaenia as well as to the absence of lymphocytosis and monocytosis in the peripheral blood. Lastly, it should be noted that the presence of a leukoerythroblastic reaction in the peripheral blood is the blood abnormality that best points to bone marrow affection by *P. brasiliensis* [274, 275].

### 10.7 Central Nervous System (CNS)

The occurrence of seizures in a patient with disseminated PCM skin lesions was the first clue pointing to possible CNS involvement [276]. Subsequent publication of several case reports showed that this disorder is more common than previously thought.

The frequency of CNS involvement varies considerably according to the methods used for investigation. Complete autopsies are not always performed, especially as concerns the spinal cord, and usually tend to correspond to terminal cases with extensive dissemination of disease. Clinical studies, in turn, do not always include neurological assessment, especially in relation to auxiliary diagnostic tests, as many patients with CNS involvement exhibit very discrete or no symptoms. One prospective study that targeted the CNS revealed symptoms suggestive of disease in 25% of the cases [277].

In general, clinical manifestations of CNS involvement occur in patients with previous or on-going involvement of the organs that are more frequent targets of PCM; however, cases with CNS involvement alone have been reported [278].

Lesions may be located in the neural parenchyma or the meninges, giving rise to two polar forms, parenchymatous or pseudotumoural, which is most frequent, and meningeal [279]. In one study, the clinical manifestations were classified as pseudotumoural in 24 of 34 analysed cases. Eleven of these cases exhibited multiple granulomas, meningoencephalitic (7/34) or meningitic (3/34) [279]; lesions were found in the brain (13/24), cerebellum (6/24) or both (5/24) [279].

Meningeal involvement may be diffuse or localised and most often affects the base of the brain. The development of disease is usually insidious and may be confounded with tuberculous meningoencephalitis. Inflammation might cause severe intracranial hypertension. Cerebrospinal fluid (CSF) abnormalities are unspecific and include mild, moderate or intense pleocytosis, with predominance of lymphocytes, and increased protein levels, with predominance ofγ-globulin, attended by decreased CSF glucose. The isolation of *P. brasiliensis* from the CSF is extremely rare.

The clinical manifestations of the parenchymatous or pseudotumoural form are highly variable as a function of the number, size and localisation of granulomas. Symptoms of intracranial hypertension predominate, which appear progressively, with signs of localisation, characterised by motor or sensory deficits, language disorders and cerebellar ataxia. Focal or generalised seizures and papilloedema have also been reported [279]. Findings on computerized axial tomography (CAT) and magnetic resonance imaging (MRI) are not pathognomonic. CAT shows ring-enhancing round lesions of variable localisations, with no signs of bone neoformation or destruction, little perifocal oedema and a discrete compression effect [280]. Assessment by MRI is better compared to CAT, particularly for lesions in the posterior fossa and when paramagnetic contrast agents are used [281]. The lesions are characterised by an iso- or hypointense T_1_ signal, a hypointense T_2_ signal, peripheral oedema and a nodular or ring contrast enhancement [281]. The authors of this MRI study found a correlation between hypointense T_2_ signal and a chronic granulomatous process. Tests performed after treatment revealed disappearance of perilesional oedema but persistence of the hypointense lesions on the T_2_-weighted scans.

Few cases of spinal cord involvement were reported, perhaps because this region is not routinely assessed on autopsy. Patients may exhibit progressive manifestations, including paraesthesia, anaesthesia and weakness in the lower limbs, faecal and urinary incontinency and neurogenic bladder presenting with episodes of urinary retention [282].

Early diagnosis of neuroPCM is based on the identification of lesions in the organs that are the more frequent targets of PCM and/or the presence of epidemiological antecedents denoting high risk of infection with *P. brasiliensis* in patients with neurological complaints

### 10.8 Urogenital System

Few cases of PCM with urogenital involvement have been reported, and the number of published case series is even smaller. Viana (1914) found kidney lesions during the autopsy of a patient with disseminated disease [245]. Urogenital lesions usually occur in cases in which other organs are also affected, are almost exclusive to males, and are seldom responsible for the patient’s main complaints. Rather, such lesions tend to be incidental autopsy findings [283]. Themost often affected structures are the epididymis, testicles and prostate, alone or in combination [284]. Pain in and increased volume and consistency of testicles and epididymis, difficulty urinating, pollakiuria and enlarged, hardened prostate are found in cases with urogenital PCM.

Urogenital involvement is rare among females, representing only 10% of cases. Despite the low frequency, cases with lesions in the ovaries and adnexa [285], placenta [286, 287] and breasts [288-290] have been reported.

### 10.9 Thyroid

Very few cases of thyroid affection by *P. brasiliensis* have been reported, being incidental autopsy findings [160, 291]. One single case of symptomatic thyroid involvement has been described; the patient exhibited a chronic condition characterised by weight loss, nervousness and neck pain, followed by restlessness, irritability, anxiety, insomnia and excessive sweating. The patient also reported neck pain radiating to the ear [292]. Physical examination revealed thyroid hypertrophy and erythema on the skin over the gland. The sample collected *via* puncture aspiration contained typical follicular cells and the characteristic *P. brasiliensis* yeast-like cells. Chest radiographs revealed lung involvement. The first study of the thyroid function in patients with PCM assessed the serum thyroxin (T4) and triiodothyronine (T3) levels and the response to thyrotropin-releasing hormone (TRH). The results showed that the serum T3 levels were low in a large number of patients, all of whom exhibited severe forms of PCM. These findings suggest that the peripheral conversion of T4 into T3 is reduced but do not point to the occurrence of any type of hypothyroidism, *i.e.*, primary, secondary or tertiary [293].

### 10.10 Eyes and Adnexa

The first report of eye affection by *P. brasiliensis* dates to 1923 [276], and until 1988, approximately 50 cases were reported, corresponding to patients with lesions in other organs [294]. Only one of the eyes is affected, with no predominance of either. Palpebral and conjunctival lesions are very frequent, while there are few reports of anterior uveitis or choroiditis [294, 295]. Palpebral lesions begin as papules, usually close to the lid margin, that then grow and develop ulcers in their centre. The ulcers’ base exhibits fine haemorrhagic points and thickened, hardened margins, evoking the moriform lesions described by Aguiar Pupo. The earliest eye lesions may mimic styes (*hordeolum*) or even bacterial blepharitis [294].

### 10.11 Other Organs

*P. brasiliensis* can involve any organ, giving rise to symptomatic or asymptomatic lesions; the latter are usually casual or incidental autopsy findings. The involvement of some organs is so rare that PCM is not even suspected, except when attended by lesions in more common sites. It was on these grounds that heart, vessel, pituitary, spleen and striated muscle involvement was detected [296]. It should be noted that the modern diagnostic imaging methods and performance of more invasive diagnostic procedures increased the frequency of confirmation of lesions in these organs.

## 11 CLASSIFICATION OF CLINICAL FORMS

The host-fungus interaction can be characterised as infection or as disease with several clinical forms.

This classification is based on criteria formulated by a group of specialists who met at the Third International Congress on Paracoccidioidomycosis, held in Medellin (Colombia) [297], with some modifications resulting from the study of the acute/subacute form [217]; the introduction of the regressive form, well-established for other systemic mycoses; a definition of the mixed [298] and the isolated organic forms [299]; and the characterisation of severity [219, 220].

***Paracoccidioides infection*** is exhibited by healthy individuals who contacted the fungus and developed an efficacious cell-mediated immune response that was able to hinder the progression of infection into actual disease. Infection is confirmed by a positive intradermal reaction to the specific antigen or identification of latent foci in the autopsy of individuals who died from other causes [83].

The ***regressive form*** is the most benign type of PCM; patients only exhibit mild clinical manifestations, usually involving the lungs, positive skin reaction to paracoccidioidin and clinical regression even without treatment [302, 303]. This form is seldom diagnosed because a lack of awareness of *P. brasiliensis*’s ecological niche does not allow correlating a suspicious contact with self-limited clinical manifestations, which are thus attributed to other causes.

The ***acute/subacute, chronic, mixed and isolated organic forms*** represent progressive disease and are characterised by signs and symptoms associated with the involvement of one or more organs. The characterisation of these forms is based on the patient’s age; duration of symptoms; clinical manifestations; the presence of associated diseases and aggravating factors; the overall state of health and nutritional status; plain chest radiographs; and skin reaction to paracoccidioidin and serum anti-*P. brasiliensis* antibodies as measured by the double immunodiffusion (DID) test.

The ***acute/subacute form (AF)*** of PCM usually affects children, adolescents and young adults, for which reason it is also known as *juvenile form*. The symptomatology develops over a short period of time, with a median of two months [198]. It is characterised by clinical manifestations that are compatible with involvement of the mononuclear phagocyte system, to wit, lymph node enlargement, hepatomegaly and/or splenomegaly, and (less often) bone marrow involvement. Lymphadenopathy occurs in several superficial and/or deep lymphatic chains and is the predominant clinical manifestation of disease. The involvement of mucous membranes is infrequent, occurring in 17 to 20% of cases, and pulmonary involvement is even rarer, being present in 5 to 10% of patients [26, 198, 304].

*P. brasiliensis* may be isolated from the bronchoalveolar lavage fluid of patients with AF with no clinical or radiological evidence of lung involvement [305]. In such cases, the lungs merely act as portal of entry.

According to the predominant manifestations, AF can be subdivided into four clinical forms [213]: a) with superficial lymphadenopathy; b) with abdominal or gastrointestinal involvement; c) with bone involvement; and d) with other clinical manifestations.

The identification of adult patients with PCM and clinical characteristics of AF is not a rare event. In such cases, the clinical form should be defined as acute/subacute, also known as *juvenile type* [297]. A recent study showed that patients under 30 years of age and with the AF of PCM exhibit a higher incidence of skin lesions, more frequent and more severe eosinophilia, and higher serum levels of precipitating antibodies (as assessed by the DID test) compared to patients over 29 [199]. These differences allow establishing a clinical-laboratory pattern of AF that manifests in children, adolescents and young adults and another pattern occurring in adults [199].

For the purpose of decision-making on treatment and prognostic assessment, AF may be classified as moderate or severe. The possibility of mild disease is never considered for this population of patients because the early and rapid development of disease and the intense involvement of the mononuclear phagocyte system are indicative of severe depression of the cell-mediated immune response.

The *chronic form (CF)* of PCM usually manifests in adults over 30 years of age and with long-lasting symptoms, *i.e.*, for more than six months. Lung involvement is the rule, although it may be absent in some cases, and involvement of the mucous membranes of the UADT is very frequent. While lymphadenopathy also occurs, it usually involves lymphatic chains of the neck in a localised manner only and is not the dominant finding.

As to its severity, CF is classified as mild, moderate or severe.

Patients with mild CF are in good state of health and exhibit a satisfactory nutritional status, and weight loss is less than 5% of the patient’s usual body weight. Lung involvement, which is very frequent in CF, is mild or may even be absent. Tegumentary involvement, especially the mucous membranes of the UADT, is discrete or absent. When present, lymphadenopathy is limited to the chains of the head and is of the inflammatory non-suppurative type. Patients do not exhibit signs of involvement of other organs or systems. The serum levels of anti-*P. brasiliensis* antibodies are low, and the intradermal reaction to paracoccidioidin is strong. Lastly, all of the suggested criteria should be met for CF to be classified as mild.

The other pole is represented by severe CF, characterised by poor overall state of health and nutritional status, with weight loss greater than 10% of the usual body weight. Patients exhibit intense respiratory symptoms and chest radiographs evidence lung involvement. When present, lymphadenopathy is not limited to the chains in the neck and is of the tumoural or suppurative type. Skin lesions are usually present and are severe. Involvement of other organs, such as the adrenal glands and CNS, is frequent. In general, patients exhibit high levels of anti-*P. brasiliensis* antibodies in the serum and negative reaction on the paracoccidioidin skin test. The presence of three of these criteria suffices to characterise the severe CF of PCM.

The moderate CF of PCM is intermediate between the polar forms just described. In general, patients have a moderately affected overall state of health and nutritional status, with weight loss equivalent to 5-10% of the usual body weight. Signs of involvement of other organs or systems, such as the adrenal glands, CNS, gastrointestinal tract and bones, are usually absent. The serum levels of specific antibodies are moderately elevated, and the response to the intradermal test with paracoccidioidin is also moderate.

The population of patients with this form of PCM is highly heterogeneous. Some patients meet almost all of the criteria for the mild form of CF, thus representing a group with moderate CF that is quite close to the mild form. Consequently, these patients may be considered as presenting a *mild-to-moderate* form of disease. Other patients meet just one or two of the criteria for severe CF, thus representing a group with moderate CF that is close to the severe form and correspondingly can be classified as a *moderate-to-severe* form. Lastly, in some patients, the severity of the clinical manifestations is equally distant from both the mild and severe forms; thus, these patients are simply classified with the moderate form of disease.

### 11.1 Mixed Forms

There are patients presenting some clinical manifestations that are typical of the AF, while other findings are proper to the CF, which makes their classification difficult. These variations of the clinical presentation of PCM should be considered as mixed forms and are seen in patients with severe depression of the cell-mediated immune response and extensive dissemination of disease; it was for this population of patients that the term “mixed forms” was first suggested [298].

### 11.2 Isolated Organic Form

In some rare cases, the clinical manifestations of PCM relate to one single organ but do not meet the criteria for either AF or CF. In these cases, diagnosis usually requires invasive methods and histopathological assessment, which often reveals involvement of some contiguous structures, most often the lymph nodes. These patients should be classified as with the isolated organic form of disease, a term already used by pathologists [299].

### 11.3 Residual Forms

(Sequelae) are very frequent in PCM because a considerable portion of patients develop sequelae. Pulmonary sequelae stand out due to their frequency, severity, and the limitations they impose on the patients’ lives and are primarily characterised by fibrosis and emphysema.

Even when treatment is appropriate, patients with Addison’s syndrome frequently require hormone replacement therapy for life. The neurological sequelae vary greatly according to the localisation of lesions but generally impose considerable limitations to the patients’ activity. Tracheal lesions cause hard-to-treat sequelae, which occasionally require surgical intervention. Lesions in the gastrointestinal tract might cause obstruction or sub-obstruction, requiring two-stage surgical correction. Skin and mucosal lesions are very often disfiguring, especially laryngeal lesions, causing considerable and often irreversible impairment of the patient’s voice. Other organs may also develop sequelae.

## 12 CLINICAL MANIFESTATIONS OF PCM CAUSED BY *P. LUTZII*

On the contrary of laboratory evaluation, studies on clinical manifestations are scarce in PCM caused by *P. lutzii*, with only two publications – two patients with chronic form [300] and one case of fatal fungemia [301]. A comparison of the clinical manifestations between patients with PCM caused by *P. brasiliensis *and cases by *P. lutzii* is ongoing in the Faculdade de Medicina – Universidade Federal de Mato Grosso do Sul.

## 13 CLINICAL MANIFESTATIONS IN SPECIAL HOSTS

### 13.1 AIDS-PCM Co-Infection

After the emergence of the AIDS pandemic in the 1980s, cases of comorbidity PCM and HIV began to be reported in PCM endemic countries, particularly Brazil, Venezuela and Colombia [298, 306]. Different from histoplasmosis and cryptococcosis, which are more commonly identified in AIDS patients, large PCM case series have shown that, in Brazil, only 4-5% of patients with PCM have comorbidity HIV infection [307-309], and approximately 1.5% of AIDS cases have comorbidity PCM [307, 310].

This relatively low prevalence has been attributed to the routine use of cotrimoxazole for *Pneumocystis jirovecii* pneumonia (PJP) prophylaxis in patients with AIDS, which may also prevent PCM [307]. However, Morejón *et al*. [307] reported that a high percentage of patients with comorbid PCM-HIV regularly used cotrimoxazole prophylaxis for the prevention of PJP.

Additionally, the epidemiological factors associated with fungi exposure could explain the low observed prevalence of *Paracoccidioides*/HIV co-infection. AIDS has been identified as more prevalent in urban centres, while PCM generally predominates in small towns with rural economies; therefore, urban AIDS patients may have less exposure to *Paracoccidioides* sp. In our previous study, in which we evaluated the prevalence of paracoccidioidical infection in HIV-infected individuals using the gp43 intradermal test, we found that 12.2% of HIV patients were co-infected with *Paracoccidioides* sp [311] and were therefore at risk of becoming ill due to the decreased cellular immunity that is characteristic of HIV infection. This prevalence was lower than that observed in rural areas of the same region, with a co-infection prevalence of 47% previously identified among rural settlers [91].

Patients with the PCM-HIV comorbidity have been reported to be younger and less involved with farming activities [306, 307], and the vast majority of these patients have CD4 counts ≤ 200cells/mm^3^. The clinical manifestations of PCM in HIV patients range from mild to severe. PCM cases with extrapulmonary organ involvement, including involvement of the lymph nodes, liver and spleen have been more frequently identified in HIV-positive than HIV-negative patients [307]. Involvement of the mononuclear phagocyte system is characteristic of acute/subacute PCM; however, in patients with chronic PCM, the lungs and mucous membranes of the upper aerodigestive tract have also been found to be affected [298, 307]. These findings suggest that these patients have a mixed form of PCM, which was included in the PCM classification proposed in 1987 [312].

Regarding the diagnosis of PCM, attention should be paid to the fact that antibody detection tests have demonstrated reduced sensitivity in cases with HIV [307]. Therefore, one should not completely rule out a diagnosis of PCM in HIV patients when serological test results are negative. In these cases, microbiological methods should be preferentially used, as diagnostic tests, such as experimental antigen-based and molecular biology tests, are not yet available for use in routine care. The combination of two methods of antibody detection, ELISA tests and either counter-immunoelectrophoresis or immunodiffusion, has shown to have increased sensitivity [313].

Antifungal treatment recommendations do not differ by HIV status; however, itraconazole treatment should be avoided in cases with tuberculosis because this antifungal agent may interact with rifampicin, thereby reducing serum itraconazole levels. Tuberculosis co-infection has previously been observed in 9.4 to 58.3% of cases with PCM-HIV comorbidity [307, 310]. Secondary prophylaxis with antifungal agents until T CD4^+^ lymphocytes levels reach at least 200cells/mm^3^ has been suggested based on studies assessing other opportunistic mycoses [177].

### 13.2 PCM-Lymphoproliferative Disorders

Studies on the association between PCM and malignant tumours are relatively scarce. Carcinomas are the most frequently diagnosed type of neoplasia in PCM patients, that has been attributed to the humoral and cellular immune dysregulation induced by *P. brasiliensis* [314]. On the other hand, the immunesupression caused by some neoplasias and/or their treatments could reactivate quiescent *P. brasiliensis* foci. Although the co-morbidity PCM-lymphoma is rare, with cases showing PCM previously to lymphoma, it is possible to suggest that the chronic stimulation by paracoccidioidal antigen could be implicated in the origen of B lymphomas [314]. Additional studies should be performed to better understand this subject.

## 14 LABORATORY FINDINGS

There are few studies on the laboratory abnormalities exhibited by patients with PCM upon admission and during antifungal treatment, and they generally involve small numbers of patients and a short follow-up period. The abnormalities detected upon admission, *i.e.*, before the onset of antifungal treatment, reflect the effects of PCM and must return to normal. In turn, the abnormalities that appear after the onset of treatment are usually side effects.

### 14.1 Complete Blood Count

This test usually shows normocytic and normochromic anaemia. Leukocytosis might be present, most often in cases with CF. Eosinophilia is the most common finding, being most frequent among cases of AF. The erythrocyte sedimentation rate is elevated in the vast majority of cases and serves as an auxiliary criterion of cure [315-317].

### 14.2 Hepatobiliary System

Abnormalities of hepatobiliary variables are more intense in AF compared to CF (Table **3**) [318]. These studies confirm the results of previous studies conducted with small number of patients with AF [217, 319].

### 14.3 Metabolic Abnormalities and Acute Phase Reactants

The changes most frequently found in both AF and CF are reductionsin serum cholesterol and albumin levels and elevated levels of α_1_-acid glycoprotein, globulins and mucoproteins. The reduction of serum cholesterol and albumin and elevation of globulin levels are more intense in AF compared to CF (Table **3**).

The elevation of γ-globulin, mucoproteins and α-1-acid glycoprotein were previously reported [320].

AF – acute /subacute form; CF- chronic form; AST-aspartate aminotransferase; ALT-alanine aminotransferase; CB- Conjugated bilirubin; TB-total bilirubin; ALP- alkaline phosphatase; γ-GT – gamma-glutamiltransferase; [a]- increase of; [d]- decrease of;... absence of information, due to the impossibility to compare continuous variables, considering the short number of patients with alteration of this parameter.

## 15 DIAGNOSIS

### 15.1 Mycological Diagnosis

Diagnosis of PCM is established based on the demonstration of *P. brasiliensis* in clinical samples [321]. The fungus is visualised under plain optical microscopy, whereby its morphology and reproduction by multiple exogenous budding, typical of the parasitic form of the fungus, allow for its identification Fig. (**1**). However, small forms might be confounded with *Histoplasma capsulatum* var. *capsulatum* or non-encapsulated strains of *Cryptococcus neoformans,* especially on histopathological examination. In such cases, culture of samples, inoculation into susceptible animals or an immunofluorescence test with fluorescein-labelled hyperimmune serum is needed.

The identification of *P. brasiliensis* in sputum is more difficult than in skin lesion scraps and lymph node materials, in which the amount of fungi is large. Initially, the technique merely involved direct observation of fresh samples on slides with covers lips. Subsequently, sputum clarification with 10% KOH Fig. (**1A**) or 4% NaOH and homogenisation were successively suggested [322]. The rate of positive results of examinations using homogenised sputum samples is much higher compared to those using cleared sputum only.

The aforementioned techniques allow for the identification of fungus in sputum samples in the vast majority of cases. According to current recommendations, mycological testing should be performed in sputum samples collected over three consecutive days, and a new sample should only be collected when initial testing is negative.

Another useful technique is the cell block Fig. (**1B**) preparation of sputum in paraffin, followed by staining of sections with haematoxylin-eosin (HE) Fig. (**1C**) and methenamine silver (Gomori-Grocott) [323, 324]. This technique allows for slides to be conserved for many years, preserving of the paraffin blocks with included sputum, and the preparation of new sections that can be stained for acid-fast bacilli or neoplastic cells. This technique is rather expensive and demands considerable time and is indicated when direct mycological examination yields negative results. It should be noted that silver staining facilitates visualising the fungus, which is a valuable help Fig. (**1D**). The sensitivity of the available methods for the investigation of *P. brasiliensis* in sputum tends to be somewhat lower for patients with lung radiological lesions of the purely interstitial type, for whom the number of samples tested should be higher.

An assessment of routine diagnostic methods at a university hospital over 34 years showed that the sensitivity of direct mycological examination for various different clinical samples was 75%, and 63% for sputum; the sensitivity of cell block preparation of sputum was 95% [325].

*P. brasiliensis* should be cultured in one of the following culture media: Mycosel (BBL) or Mycobiotic Agar (Difco), SABHI (Difco), Sabouraud agar oryeast extract agar. Sputum samples should be digested with pancreatin or N-acetyl-L-cysteine and then is inoculated into plates or tubes in appropriate culture media at room temperature. Transformation of the filamentous into the yeast-like phase, characteristic of *P. brasiliensis*, is obtained by inoculating the fungus onto Kelley medium with haemoglobin at 35-36ºC.

Mycological examination can also be performed using tissue fragments ground in a sterile gral and then placed on a slide under a covers lip or cut with a razor blade and inoculated into the culture medium.

### 15.2 Histopathological Diagnosis

Fragments of tissue collected through biopsy and stained using the H&E and methenamine silver methods are used to establish the histopathological diagnosis of disease. H&E staining allows for assessing the host’s inflammatory response, the organisation of granulomas, and the presence of the typical *Paracoccidioides* sp yeastcells. In turn, methenamine silver stains the fungus wall, thus revealing the presence of fungi with characteristic exogenous sporulation that gives rise to the so-called “mickey mouse” form, strongly suggestive of *P. brasiliensis*. In addition, the “steering wheel” form can be observed, which is pathognomonic for this fungus species. Methenamine silver staining does not allow for assessing the inflammatory response in tissues. Histopathological examination has a sensitivity of 97% for the diagnosis of PCM [325].

Histopathological examination allows both establishing a diagnosis of PCM and determining its severity according to the type of granulomas that are present – compact in patients with preserved cell-mediated immunity and loose among patients with severe depression of cell-mediated immunity.

### 15.3 Serological and Molecular Diagnosis

#### 15.3.1 Antibody Investigation

*General considerations.* Serological tests for the detection of anti-*P. brasiliensis* antibodies are helpful for diagnosis of PCM and represent indicators of severity as well as criteria for monitoring the response to specific treatment. The diagnostic value of the various serological tests depends on their accuracy, which is strictly related to the antigens used and the selected cut-off point. Yeast-like cell culture filtrate is the antigen used in gel precipitation tests because it exhibits satisfactory diffusion in the gel. Antigen-antibody complexes precipitate due to their high molecular weight, forming a macroscopically visible line. In turn, the polysaccharide antigen is used in complement fixation test (CFT) because it exhibits satisfactory fixation of the complement system, which is the basis of this test.

Along the last decades we have observed the preparation of *Paracoccidioides* sp antigens in different Services (*in house*), without a standardized protocol, leading to innumerable problems, such as reproducibility and repetitiveness [326]. As a consequence, results obtained in different centers can hardly be compared. In addition, the identification of different species - *P. brasiliensis* and *P. lutzii* and the *P. brasiliensis* cryptic species has suggested the use of antigens prepared from the dominant species in the region the patients come from. It is another difficulty for the appropriate interpretation of the serological tests [327, 328].

Possible cross-reaction with antibodies in the serum of patients with other diseases, fungal or not, is a permanent source of concern. This problem can be minimised by standardising the antigens used.

The identification of the *P. brasiliensis* 43-kDa glycoprotein (gp43) represented a significant contribution to the knowledge of the immune response in PCM [19]. Consequently, any antigen formulation adequate for serodiagnosis must include this molecule [329]. However, some *P. brasiliensis* isolates do not express gp43 [330]. *P. brasiliensis* cultures are composed of various clones, most of which do not initially secrete gp43. Only after 10 subcultures do these isolates begin to express the gene related with the secretion of this glycoprotein, albeit in a variable manner [331].

Aiming to improve the serological diagnosis of PCM, much effort has been devoted to avoid the use of crude antigens and replacing them with recombinant proteins [332, 333], which in many cases reduce the frequency of cross-reactions [334]. However, the production of these molecules is expensive, which limits their use at research centres [335].

Antibody isotypes to a *P. brasiliensis* somatic antigen were evaluated by ELISA in PCM patients with AF and CF, who showed no differences in total IgG, IgG_1_, IgG_2_, or IgG_3_. Higher levels of IgG_4_ were observed in AF patients, while IgA predominated in CF patients. However, these isotypes have not been routinely assessed in the diagnosis and treatment follow-up of PCM patients.

*Tests used.* The main tests developed for diagnosis of PCM are CFT, agar gel precipitation tests (DID, and counter immunoelectrophoresis – CIE), indirect immunofluorescence (IIF), immunoenzymatic tests (ELISA, magnetic ELISA - MELISA, inhibition ELISA), dot-blotting, and western blotting.

The sensitivity of CFT varies from 69.5 to 93%, and its specificity ranges from 96.7 to 100%, the predictive values being over 90% [336, 337] (Table **4**). However, CFT stopped being used because it is difficult to perform, requires large amounts of reagents [22, 338], and is characterised by frequent cross-reactions [339] (Table **4**).

DID for diagnosis of PCM was introduced by Restrepo [23]; the values of the accuracy parameters are high, and the test results correlate with efficacious treatment [340]. DID is the most widely used serological test for the diagnosis of PCM, being considered as the standard by the authors of the Manual of Epidemiological Surveillance [340]. CIE with antigens extracted from a sonicated cell suspension exhibits a sensitivity of 77%, a specificity of 95%, and an accuracy of 94% (Table **4**) [341].

Although simple to perform, the latex agglutination (LA) slide test is scarcely used. Its sensitivity is lower compared to the gel precipitation tests (DID and CIE) but has a negative predictive value of over 80% (Table **4**). The formulation with crude antigen should be preferred over the formulation with an ethanol-treated antigen because the former is easier to prepare and exhibits less frequent cross-reaction with tuberculosis (Table **4**), the main differential diagnosis of PCM [342]. One study assessed the LA test using a pool of crude fungal exoantigens and found a sensitivity of 84% and a specificity of 81%; however, a cross-reaction with aspergillosis and histoplasmosis was observed (Table **4**) [343].

IIF was also used for diagnosis of PCM [344]; as advantages, it can be used with anti-complementary sera [345] and allows for characterisation of the isotypes of anti-*P. brasiliensis* immunoglobulins [24]. Despite its high sensitivity, IIF exhibits frequent cross-reactions, especially with sera from patients with histoplasmosis [346] (Table **4**). The need for a fluorescence microscope, subjective readings, and manual steps are limitations of this method [347].

ELISA affords several advantages. Specifically, the reagents last a long time when they are safely stored, and the test is simple to perform, provides quantitative results, and has high sensitivity. The test can detect antibodies in concentrations as low as 0.05μg/ml [345]. However, its sensitivity and specificity vary according to the antigen used and the selected cut-off point.

Using *P. brasiliensis* yeast-like cell culture filtrate as the antigen and a titre of 1:80 as cut-off point, the sensitivity and specificity of ELISA reached 100% [348]. However, it exhibited cross-reactions with histoplasmosis, cryptococcosis, aspergillosis and lacaziosis (lobomycosis) (Table **4**). In one study that employed cytoplasmic antigens, 66% of the tested sera reacted at titres equal to or higher than 1:128, and the specificity of the test was 95%. However, this test exhibited cross-reaction with histoplasmosis (36%) (Table **4**) [349].

The reaction of antibodies from PCM patients with gp43 predominantly involves peptide epitopes (>85%) [350]. The cross-reaction of serum antibodies from patients with histoplasmosis or lacaziosis has been attributed to galactose-containing carbohydrate epitopes in N-linked carbohydrate chains of gp43. This occurs when methods using plastic-immobilised antigen (e.g., ELISA) are performed. ELISA is easy to perform and presents high sensitivity, but low specificity. Never the less, the improvement of its specificity using sodium metaperiodate antigen (gp43) treatment, serum absorption process with *Candida albicans* or *Histoplasma capsulatum* antigens, and dilution of serum galactose showed poor results [351].

In spite of several immunological methods useful for PCM diagnosis, many of them are time-consuming, lack accuracy, are expensive, result in cross-reactions or are unavailable for routine clinical laboratories, justifying the proposal of new tests. In this regard, dot-blotting is a practical, rapid and less expensive method (Table **4**) [352, 353].

Dot-ELISA method has been proposed to detect anti-*P. brasiliensis *antibodies in sera from patients with PCM using membranes impregnated with fungal antigens. It has advantage to be used by laboratories with few resources or even directly in the field. In addition, it presents high values of the accuracy parameters [354] (Table **4**).

Western blotting or immunoblotting have been employed to detect anti-*P. brasiliensis* antibodies in sera from patients with PCM using membranes impregnated with fungal exoantigens. Using gp43 and gp70, the sensitivities for diagnosing PCM were 100% and 96%, respectively. However, this test exhibited cross-reactions with sera from patients with histoplasmosis (Table **4**). Although immunoblotting has high sensitivity, this test has not been standardised as routine practice in most laboratories [355].

*Diagnosis of P. lutzii.* The investigation of antibodies in sera from patients with PCM likely caused by *P. lutzii* was assessed using three methods and three types of antigens obtained from strain EPM 2008 [356]. The best results were achieved by the DID test and using cell free antigen (CFA), with sensitivity, specificity, positive and negative predictive values and accuracy of 100%.

#### 15.3.2 Antigen Investigation

Antigens are investigated using known specific antibodies to detect antigen-antibody reactions. As an advantage, there is no dependence on antibody production by the patient, which is impaired in immunosuppressed cases.

In one study, inhibition ELISA using BALB/c mice monoclonal IgG antibodies targeting an 87-kDa protein obtained from *P. brasiliensis* yeast cell culture filtrate had a sensitivity of 80% and a specificity of 100%; however, the frequency of cross-reaction with sera from patients with aspergillosis, histoplasmosis, cryptococcosis and sporotrichosis was high [357] (Table **5**). In another study, an investigation was made of antigens in the serum, bronchoalveolar lavage fluid and CSF from patients with PCM. Using anti-gp43 and anti-gp70 monoclonal antibodies, a sensitivity of 90 to 96% and a specificity of 96 to 100% were observed [358] (Table **5**). Despite the success of this method in both the diagnosis and follow-up of patients after the onset of treatment, its viability for use in the clinical routine is unknown [359].

In one study, the detection of circulating *P. brasiliensis* gp43 and gp70 in the CSF of patients with neuroPCM had a sensitivity of 90.9% and a specificity of 100% (Table **5**) [360].

In another study, *P. brasiliensis* antigens were detected in the urine of patients with active PCM before the onset of antifungal treatment, with the 43- and 70-kDa antigens being the most frequently observed antigens [361]. Antigen investigation was also useful for the monitoring of treatment given that reactivity decreased in the samples collected during clinical recovery and increased in the case of relapse.

Excellent article review was recentily published focusing the different methodologies for the identification of specific antibodies and antigens used in serological diagnosis of PCM [362].

#### 15.3.3 Molecular Diagnosis

Molecular methods allow for confirming the aetiology of disease through the identification of DNA fragments specific to *P. brasiliensis*, with no need to culture the fungus. Among these methods, polymerase chain reaction (PCR) and nested PCR stand out.

Nested PCR was employed to detect *P. brasiliensis* DNA fragments through the use of primers derived from the gene that encodes gp43 and detection of a 196-base pair (bp) sequence [363].

The use of molecular markers is important for diagnosis of PCM as well as for ecological, molecular and epidemiological studies of *P. brasiliensis* in Latin America. In this regard, the 5.8S and 28S ribosomal genes and intergenic regions of *P. brasiliensis* (Pb01) were amplified and sequenced; these products were able to distinguish *P. brasiliensis* from other pathogenic fungi on PCR [364].

In a clinical study that used specific primers designed from a 0.72-kb fragment, *P. brasiliensis* DNA was detected in the sputum and CSF of patients with PCM [365].

A PCR assay that employed primers derived from the gene that encodes gp43was able to detect *P. brasiliensis* DNA in sputum; the technique had a detection limit of 10 cells/mL of sputum [366].

One study assessed sera from patients with suspected PCM by means of conventional PCR using primers for the ITS1 ribosomal DNA of *P. brasiliensis*. The results showed that this technique was not effective for detecting *P. brasiliensis* DNA in serum [367].

Although PCR and nested PCR are highly sensitive, specific, or rapid methods, they are still not available for routine diagnosis of PCM, especially in the countries where the disease occurs, *i.e.*, developing countries.

#### 15.3.4 Diagnosis of Relapse

Relapse is defined as the reappearance of PCM in patients who remained in a state of clinical, radiological, and serological cure for two years after discontinuation of antifungal treatment [368].

The DID test has low sensitivity for diagnosis of relapse (45%). ELISA should therefore be used, exhibiting a positive result in 80% of cases. In addition, the hypothesis of relapse cannot be ruled out without judicious mycological assessment [368].

## 16 CASE DEFINITION

PCM cases are defined as follows: a) confirmed cases, characterised by the presence of suggestive clinical manifestations and detection of typical of the *P. brasiliensis* yeast forms in clinical samples; and b) probable cases, characterised by the presence of suggestive clinical manifestations and detection of specific antibodies in the serum by the DID test, but without identification of *P. brasiliensis* in clinical samples [177]; c) possible: patient with at least one of the following clinical manifestation with a 4-week duration, after exclusion of tuberculosis and other diseases with similar symptomatology: 1) cough with or without expectoration and dyspnoea; 2) sialorrhea, odynophagia, hoarseness. 3) any type of skin lesions; 4) cervical or generalised lymphadenomegaly; 5) child or young adult with hepatosplenomegaly and/or abdominal mass [177].

## 17 TREATMENT AND CONTROL OF CURE

From its initial report by Adolfo Lutz in 1908 [1] until the 1940s, PCM was considered to be a fatal disease because of the lack of appropriate therapeutic agents. Since the 1940s, various drugs have been used to treat this disease with satisfactory results. The first of these agents were sulfanilamides, such as sulfapyridine [369], which were followed by the introduction of amphotericin B in 1958, which is a powerful broad-spectrum antifungal agent. Although this drug is efficacious in the treatment of PCM [370], it is quite difficult to manage because of its side effects and the need for long hospital stays for intravenous administration. Then, the efficacy of the trimethoprim-sulfamethoxazole combination (cotrimoxazole, CMX) was demonstrated, and became widely used in the treatment of PCM [371]. The development of imidazole derivatives at the end of the 1970s, particularly ketoconazole (KTC) [372, 373], further broadened the scope of therapeutic options. Itraconazole (ITC), a triazole-derivative, was introduced at the end of the 1980s and proved to be 100 times more active **in vitro** against *P. brasiliensis* than KTC [374]. Regarding to its efficacy and safety, ITC has become widely used [375-377]. More recently, voriconazole (VRC), a second-generation triazole derivative, proved to be as effective as ITC in the treatment of PCM; however, this treatment has been reported to be less safe [378]. Table **6** presents the drugs and dosages used to treat PCM.

## 18 CRITERIA OF CURE

There are four criteria of cure of PCM: clinical, mycological, radiological and immunological [379].

### 18.1 Clinical Cure

A patient is considered to have achieved clinical cure when the signs and symptoms of disease are no longer present, and normalization of the erythrocyte sedimentation rate (ESR). The normalization of the ESR was included in the criteria of clinical cure to count with an objective parameter, since the clinical evaluation can incorporate some degree of subjectivism.

### 18.2 Mycological Cure

Defined as negative results on mycological investigation for *P. brasiliensis* occurring after efficacious treatment in the materials where it had been previously detected.

### 18.3 Radiological Cure

This criterion concerns the radiological assessment of the lungs, as approximately 80% of patients exhibit the chronic form of PCM, in which lung involvement is almost always present. Radiological cure is attained when the radiological pattern becomes stable after treatment, *i.e.*, the same scar lesions are found on five radiographs taken every three months in the course of one year.

### 18.4 Immunological Cure

Immunological assessment includes evaluation of both humoral immunity, through measurement of serum anti-*P. brasiliensis* antibodies, and cell-mediated immunity.

The serum levels of specific antibodies decrease after the onset of treatment to become undetectable on the DID test or stabilise at a very low concentration on the complement fixation test (CFT). This stabilisation is considered to be a serological scar.

Cell-mediated immunity is seldom investigated after the onset of treatment [184, 380], for which reason no test is suggested as routine.

### 18.5 Apparent Cure

This notion applies to patients with clinical, mycological, radiological and immunological cure for a two-year period without receiving complementary treatment. The term *apparent cure* should be preferred to *cure* to avoid the idea that a radical cure took place, *i.e.*, that the fungus was eradicated from the body. Radical cure cannot be confirmed because foci with latent fungi certainly remain in the body after efficacious treatment.

## 19 TREATMENT REGIMEN

In the past, PCM treatment was divided in two phases: initial treatment, in which amphotericin B was used until clinical cure was achieved and/or cumulative dose limits were reached, and complementary treatment, in which sulphonamides derivatives were initiated and used until serological cure was achieved. Currently, both azoles and CMX may be used throughout the course of treatment. However, this treatment is also divided into two phases because the follow-up period differs from the initial treatment period. During the initial treatment period, patients are submitted to clinical, serological, haematological, biochemical and radiological evaluations once a month until they achieve clinical cure and normal erythrocyte sedimentation rate. During the complementary treatment, patients are submitted to clinical, serological and radiological evaluations every 3 months until one year has passed since serological cure was achieved Fig. (**9**) [381].

In the last decade, ITC and CMX were the main drugs used to treat PCM. Then, the Brazilian Guidelines for PCM (2006) indicated ITC as the first choice for treatment [177]. However, at this time, only one unpublished study has compared these two treatments, suggesting that ITC and CMX had similar efficacy; however, the duration of treatment was lower in patients who received the ITC [382]. A study performed with 200 PCM patients to identify factors predicting cure showed that 86.4% of those treated with ITC were clinically cured, while only 51.3% of those who received CMX achieved this outcome [382]. However, all the severe patients were treated with CMX, a fact that may explain the difference in efficacy.

During the same year, a quasi-experimental study with 177 PCM patients was carried out comparing ITC (n=47) and CMX (n=130) in the treatment of PCM [381]. The groups were homogenous in their epidemiological, clinical and serological characteristics. During the initial treatment, ITC and CMX presented similar effectiveness (96% and 94%, respectively). However, the time to reach clinical cure was shorter in the ITC group than in the CMX group, especially in patients with the chronic form of the disease. Additionally, in the complementary treatment, no differences in effectiveness were observed between the ITC (73% effective) and CMX (70% effective) groups. Patients with the chronic form of PCM who received ITC presented serological cure, on average, at six months of treatment, while those CMX-treated showed an average time of 17 months; however, this difference was not statistically significant, which was probably due to small number of patients in the ITC group. The rate of clinical side effects, especially epigastric pain, was higher in patients treated with CMX than in patients treated with ITC.

These studies support the indication of ITC as the first choice treatment for PCM. However, there are some situations in which CMX is preferred, including patients with central nervous system involvement - ITC is not able to cross the blood brain barrier, and patients with gastrointestinal tract involvement, as ITC has erratic absorption in the gastrointestinal tract. In countries where intravenous ITC is not available, such as Brazil, CMX may be a viable option for the treatment of severe PCM cases.

The optimal criterion for the discontinuation of antifungal treatment seems to be negative DID results, that have been identified to be correlated with the recovery of specific cellular immune responses [184]. Despite efficacious antifungal therapy, latent *Paracoccidioides* sp foci, with viable fungal cells, may remain in human tissues, requiring appropriate cellular immune responses to contain the paracoccidioidal proliferation. In a study of PCM follow-up and treatment discontinued when antibody serum levels regressed to 1:2, the relapse rate was 12% [383]. However, in a study of 400 PCM patients who discontinued antifungal therapy when negative serum DID results had been observed for at last one year, a 5% relapse rate was observed [368].

## 20 CHOICE OF DRUGS FOR INITIAL TREATMENT

The factors to be considered in the selection of the drug to be used for initial treatment of PCM include severity of disease, history of possible resistance to the antifungal agent previously used, possibility of gastrointestinal absorption of the drug, the presence of associated diseases, and patient compliance to the suggested regimen.

Severe cases should be treated with the most efficacious drug, preferentially *via* an intravenous route, at least at the beginning of treatment, to ensure high bioavailability of the drug.

Drugs that are administered by the oral route should be avoided in patients with abdominal lymphadenopathy, even when no malabsorption syndrome is evident.

The presence of associated diseases should be considered to prevent the occurrence of severe side effects. For instance, amphotericin B (AmB) should be avoided in patients with impaired kidney function and older adults with peripheral artery disease. Azole derivatives, in particular KTC and CMX, are hepatotoxic and thus must be used cautiously in patients with liver disease. As the incidence of alcoholism is high among patients with PCM, the liver biochemistry should be monitored when these drugs are used.

In addition, patients with other associated diseases also use other medications, and thus, the possibility of drug interactions should be considered. For instance, this is the case with patients receiving therapy for tuberculosis and KTC for PCM. Rifampicin stimulates the degradation of KTC, with consequent reduction of its serum concentration, eventually to levels below those needed for antifungal activity. In such cases, the dose of KTC should be increased or the drug replaced by CMX or AmB.

Drugs that were previously inefficacious for PCM in a given patient must not be used in the same patient. However, it is important to bear in mind that PCM patients sometimes exhibit poor adherence to treatment or drop out, thereby not representing true instances of drug resistance.

Sulphadiazine must be taken every six hours, which makes compliance for appropriate treatment difficult, often resulting in serum drug concentrations that are below those needed and consequent treatment failure [384].

Although a rare occurrence, PCM can affect pregnant or breast feeding women. Azole derivatives are contraindicated under these circumstances, while sulphonamide derivatives are contraindicated starting on the last month of pregnancy, as they might cause kernicterus. For this reason, AmB is the first choice for treatment of pregnant women; this drug is not teratogenic, even though it crosses the placental barrier [385].

## 21 SIDE EFFECTS

Few studies assessed hepatobiliary variables after onset of treatment with ITC. Some studies did not find any changes [376-378], while others did but without reporting their incidence or intensity [375].

A recent study conducted with 200 patients found that the incidence of elevated hepatobiliary biochemistry after the onset of antifungal treatment with CMX or ITC was higher in AF compared to CF. The pattern of abnormalities varied according to the antifungal compound used, being hepatocellular for CMX (64.7%) and mixed/mild for ITC (60.0%). The progression of these abnormalities also differed regarding to the antifungal compound used, returning to normal with CMX but persisting among patients treated with ITC. These abnormalities, however, did not demand (require) the discontinuation of the treatment in any case [318]. Gastric discomfort occurred more frequently in the group treated with CMX compared to ITC [381].

Transient elevation of the serum uric acid levels was more frequent among the patients treated with ITC (11.5%) compared to CMX (3.3%). The patients also exhibited transient reduction of the serum calcium (ITC=42.8% and CMX=21.3%) and phosphorus (ITC=23.3% and CMX=2.5%) levels [318].

## 22 TREATMENT CONTROL

In general, clinical cure occurs in a relatively short time and gives patients the false impression that they are fully healed. For this reason, they should be made aware of the risk of recrudescence and of the consequent need for prolonged treatment and periodical assessment Fig. (**10**).

Mycological cure means that the fungus is no longer present only in the materials where it had been previously detected. Appropriate methods applied by experienced mycologists are necessary for mycological cure to be safely established. Mycological cure occurs even earlier; the amount of fungi observed on direct examination decreases progressively until they are no longer found. Healing of the mucosal and skin lesions and reduction of expectoration contribute to make *P. brasiliensis* no longer present in samples.

The alveolar lesions disappear more rapidly compared to interstitial lesions, which regress slowly Fig. (**6**). Interstitial lesions exhibit variable behaviour; the smaller nodules disappear with treatment, while the larger ones persist, even after the disappearance of the respiratory clinical manifestations and serum anti-*P. brasiliensis* antibodies become undetectable. The most frequent residual disorders are pulmonary fibrosis and emphysema, appearing as fibrotic streaks and nodules and diffuse or bullous emphysema.

The decrease of the antibody serum levels determined by agar gel precipitation tests, observed in patients after treatment, correlates with alterations of the secretion of cytokines - increase of IL-2 and IFN-γ and reduction of IL-10. Based on these findings, the evaluation of the humoral immunity has been used as indicative of improvement of the cell mediated immunity [184].

The lymphoproliferative response, intradermal reaction to paracoccidioidin and balance between Th_1_ and Th_2_ cytokines return to normal after successful treatment [184, 380]. A study conducted with patients with the chronic form of PCM showed that the recovery of the cell-mediated immunity, as assessed through the quantification of mononuclear cell subpopulations and functional tests, only occurred when the patients exhibited apparent cure [380]. This correlation allows using apparent cure as a criterion of recovery of the specific cell-mediated immunity responsible for keeping the surviving fungi in the latent state.

## 23 IMMUNOSTIMULANTS

Immunostimulants were first tested for treatment of PCM in an animal model with highly satisfactory results [386, 387]. However, a single study assessed the progression of patients with PCM given antifungal agents and β-glucan as an immunostimulant.β-glucan is β-1,3-polyglucose extracted from *Saccharomyces cerevisiae* and was used in a 10mg dose, per intravenous or intramuscular routes, once per week during the first month and then monthly for one year. The patients treated with a combination of β-glucan and an antifungal agent exhibited better progression in terms of clinical manifestations, return to the normal erythrocyte sedimentation rate, and humoral and cell-mediated immunity compared to those not receiving immunostimulation [388].

β-glucan was found to behave as a powerful inducer of the production of tumour necrosis factor alpha (TNF-α) and interferon-gamma (IFN-γ) in BALB/c mice; these findings may account for the adjuvant effects of β-glucan in the treatment of PCM [389]. For this reason, β-glucan should be indicated for treatment of severe forms of disease, provided the patient’s TNF-α levels can be monitored, as it is harmful to patients in excess.

## 24 PROPHYLAXIS

The lack of knowledge regarding the ecological niche of *Paracoccidioides* sp hinders the development of prophylactic measures that are likely to prevent infection among the population most exposed to the fungus.

## 25 DISCUSSION

Perhaps the only measure with some practical value for this population is the recommendation not to use plant leaves for anal hygiene. While this practice does not prevent inoculation with the fungus, which is highly improbable, it seeks to avoid the fixation of fungi occasionally present in the bloodstream after having been inhaled. The reason is that the sequelae derived from anal lesions may be very severe, especially when they extend to the rectum.

## CONCLUSION

Investigators and laboratory technicians who work with *Paracoccidioides* sp samples should be very careful when handling culture media and infecting experimental animals. In case of accidents likely to result in infection, the involved area should be immediately washed with soap and water. In addition, the individual should be subjected to investigation for serum specific antibodies by means of DID and receive itraconazole 200mg, one single daily dose after breakfast, for one month. If no clinical manifestations appear, namely, lesions on the probable site of inoculation attended by regional lymphadenopathy, nor seroconversion occurs, the medication should be discontinued, and the patient subjected to clinical and serological assessment for an additional two months. The absence of clinical manifestations and persistent negative serology allows discontinuance of monitoring. Conversely, the presence of PCM lesions or seroconversion indicates that antifungal treatment should be maintained and the patient should be assessed and monitored according to the instituted regimen.

## Figures and Tables

**Fig. (2):**
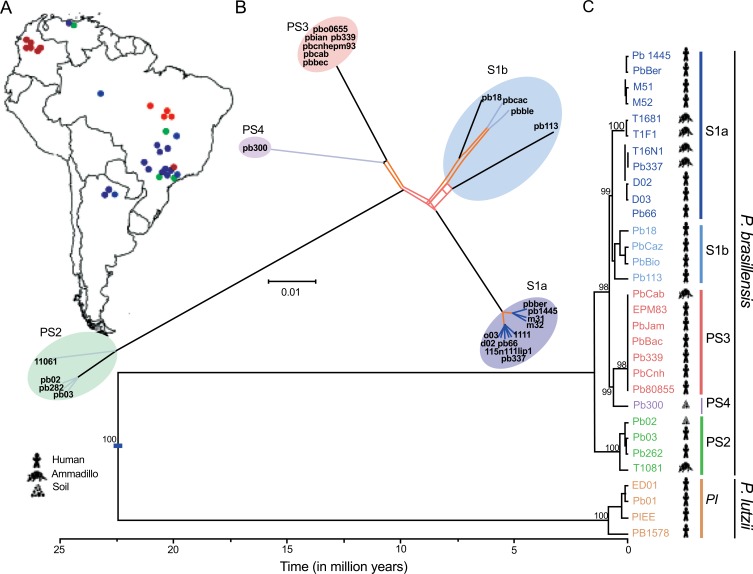
Phylogeny and recombination in *Paracoccidioides*. Two methods were used to examine strain relationships originating from across South America (A): using 614,570 SNPs, including a phylogenetic network constructed with SplitsTree4 (B), and a Bayesian calibrated phylogeny constructed with BEAST (C); bootstrap values from maximum likelihood phylogeny constructed with RAxML were included for major subdivisions. Both methods show evidence of five distinct lineages in *P. brasiliensis*: S1 (blue), which is divided into two groups S1a (dark blue) and S1b (light blue), PS2 (green), PS3 (red), and the recently described PS4 (purple). Also, this phylogeny supports the divergence between *P. brasiliensis* and *P. lutzii* (*Pl* [orange]) as a different species. In addition, the phylogenetic network of *P. brasiliensis* suggests patterns of recombination (red branches). **Note:** from Muñoz JF, Farrer RA, Desardins CA, *et al*. Genome diversity, recombination, and virulence across the major lineages of *Paracoccidioides*. 2016. mSphere 1(5): e00213-16.

**Fig. (3):**
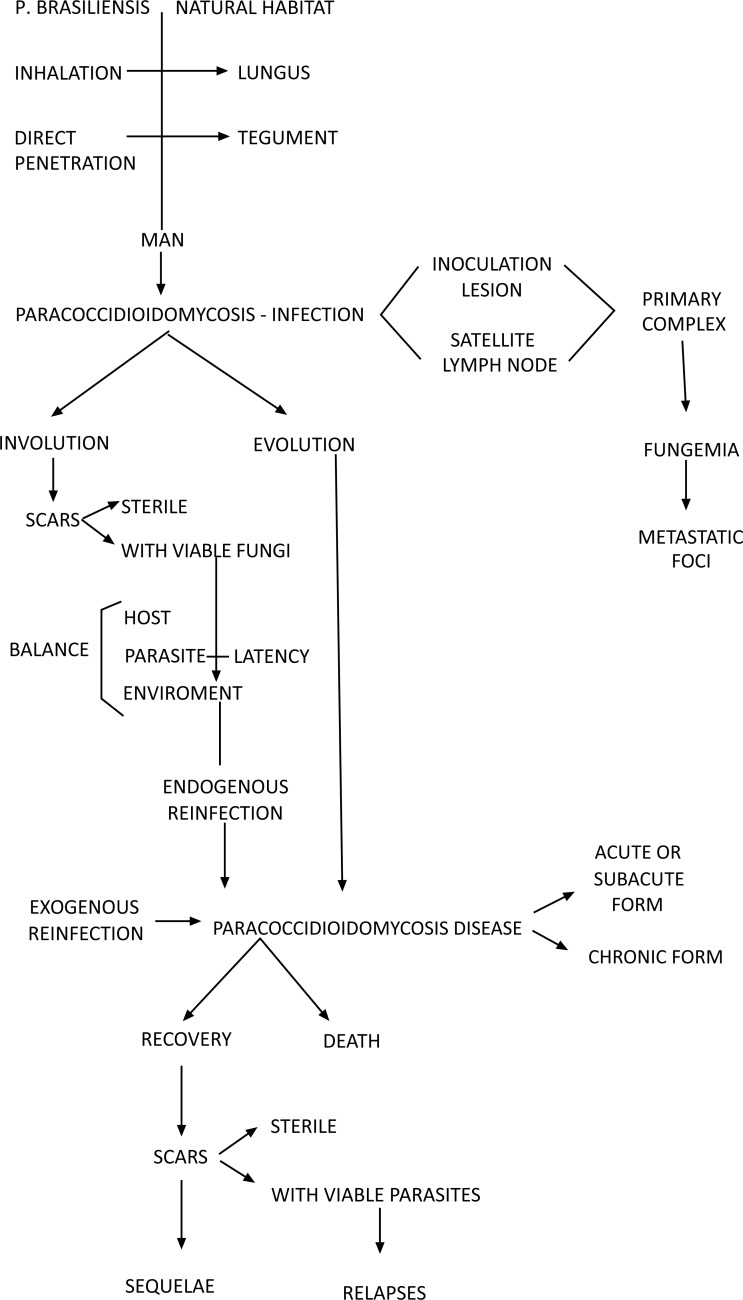
Natural history of paracoccidioidomycosis. **Note:** from Franco M, Mendes RP, Moscardi-Bacchi M, Rezkallah-Iwasso M, Montenegro MR. Paracoccidioidomycosis. Baillière’s Clin Trop Med Commun Dis. 1989; 4.1: 185 – 220.

**Fig. (4):**
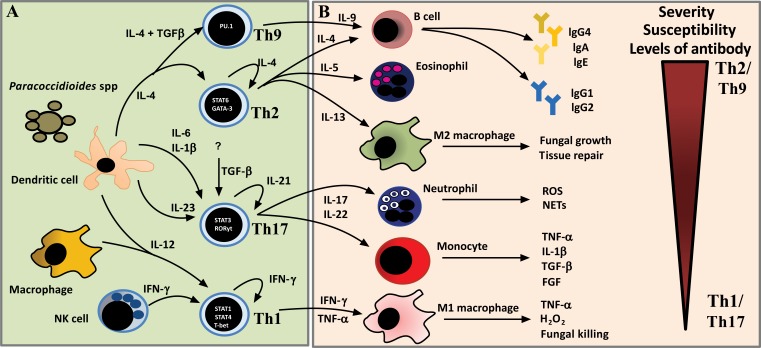
Overview of the polarization of adaptive immune in PCM.(A) Activation of T helper (Th) subsets. *Paracoccidioides* antigen-primed dendritic cells (DCs) migrate to the lymph node where they present processed antigens to naïve Thcells that differentiate into one of Th lymphocyte subsets (Th1, Th2, Th9 and Th17) depending primarily on cytokines present in the extracellular environment. For Th1 polarization, IL-12 from DCs and macrophages, and IFN-γ from NK cells activate STAT1/STAT4 signaling to induce expression of the Th1-specific transcription factor, T-bet. For Th17 polarization, IL-6, IL-1β, TGF-β and IL-23 are required to induce expression of the Th17-specific transcription factor, RORβτ, through STAT3 signaling. For Th2 polarization, IL-4 from DCs activates STAT6 signaling to induce expression of the Th2-specific transcription factor, GATA-3. For Th9 polarization, IL-4 and TGF-β are required to induce expression of the Th9-specific transcription factor, PU.1. (B) Effector phases of T cell responses. Th1, Th17, Th2 and Th9 clones could be distinguished mainly by the cytokines produced by the cells. Th1 cells release high amounts of IFN-γ and TNF-α that classically activate macrophages (M1) resulting in fungal elimination. Th17 cells secrete IL-17 and IL-22 that recruit neutrophils and monocytes. Neutrophils cells act by generate reactive oxygen species (ROS), release of neutrophil extracellular traps (NETs) that result in fungal elimination. Monocytes have been studied in PCM by induce high levels of inflammatory cytokines, such as TNF-α and IL-1β, and growth factors, such as TGF-β and fibroblast growth factor (FGF). Th2 show several functions that depend of each secreted cytokine. IL-4 induces activation of B cells and subsequent production of immunoglobulins; IL-5 triggers recruitment of eosinophils; and IL-13 is involved in the deactivation of macrophages, termed “alternatively activated macrophages” (M2), that results in fungal growth and also in tissue repair. Th9 release IL-9 and IL-21 that act in synergy with Th2 to produce antibodies.

**Fig. (5):**
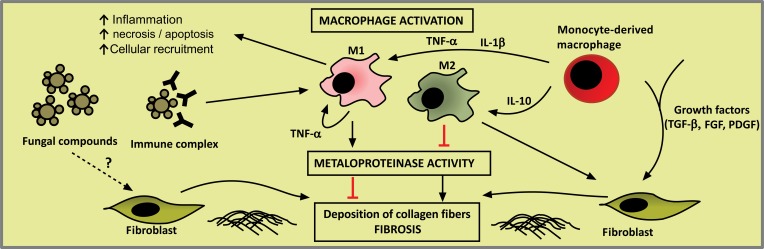
Proposed overview of fibrogenesis in PCM. The uncontrolled deposition of collagen fibers (types I and III) during a reparative and/or reactive process is recognized as fibrosis and the fibroblasts are the main cell involved in the production of collagens upon stimulation. During prolonged chronic inflammation, macrophages are classically activated (M1) by Th1 cells, pro-inflammatory cytokines and/orimmunocomplexes. These cells show intense production of cytotoxic metabolites to kill pathogens, release pro-inflammatory cytokines, induce tissue necrosis, and recruit phagocytes. Fresh monocytes from peripheral blood and monocyte-derived macrophages release inflammatory cytokines and growth factor that result inmacrophage activationsand fibroblast differentiation. Simultaneously, due to the constant tissue damage, others macrophages are alternatively activated (M2) to stimulatetissue repair. These cells promote the elimination of cellular debris, activation of fibroblast and down-regulate the activities of metalloproteinases, enzymes implicated in extracellular matrix/collagen degradation, promoting deposition of collagen fibers. On the other hand, M1-polarized macrophages up-regulate metalloproteinase activities, resulting in less collagen deposition. Besides the non-regulated, constant and prolonged reparative and reactive processes involved in fibrogenesis, do not rule out the action of molecules of *Paracoccidioides* in the activation of fibroblast.

**Fig. (6):**
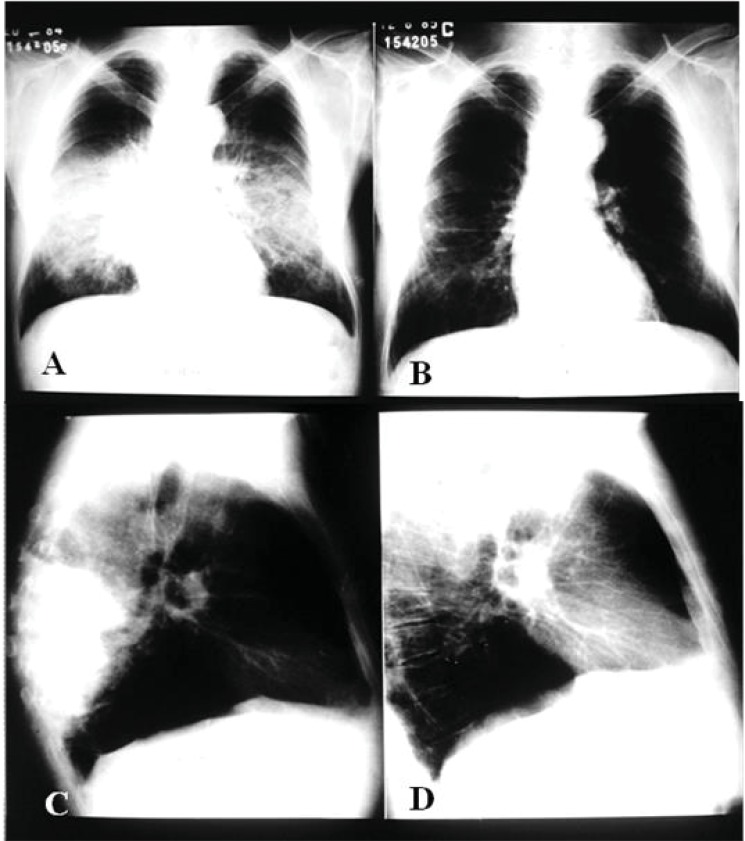
Chest radiograph of a patient with the chronic form of paracoccidioidomycosis, in antero-psterior and laterak view, showing bilateral and symmetrical alveolar lesions before (A and C) and after (B and D) treatment.

**Fig. (7):**
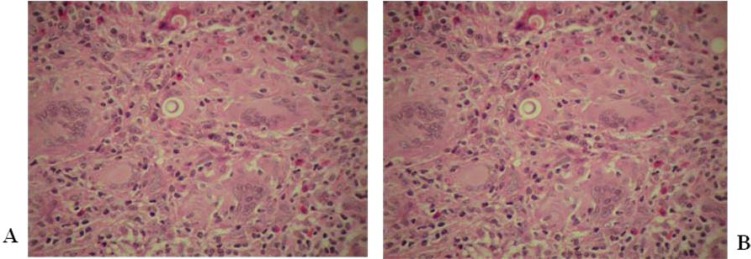
A - Paracoccidioidomycosis: infiltrated erythematous and well demarcated lesions on face; B - Paracoccidioidomycosis: compact granuloma with epithelioid and giant cells. A fungal cell is well observed in the cytoplasm of a giant cell (HE x40).

**Fig. (8):**
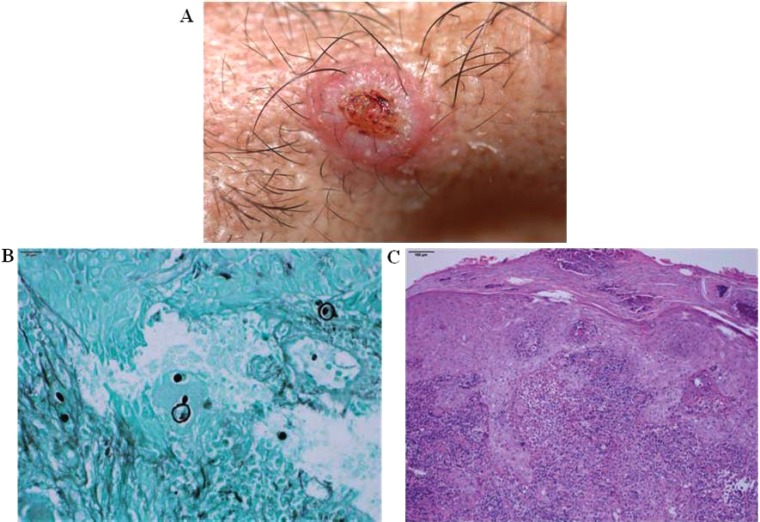
A - Paracoccidioidomycosis: ulcerated lesion with hemorrhagic dots and elevated borders on the face; B - Paracoccidioidomycosis: fungal cells stained in black, some in active multiplication. (Grocott-Gomori x 40); C - paracoccidioidomycosis: marked pseudoepitheliomatous hyperplasia with dense inflammatory infiltration in the dermis. Fungal cells can be observed. (HE x10).

**Fig. (9):**
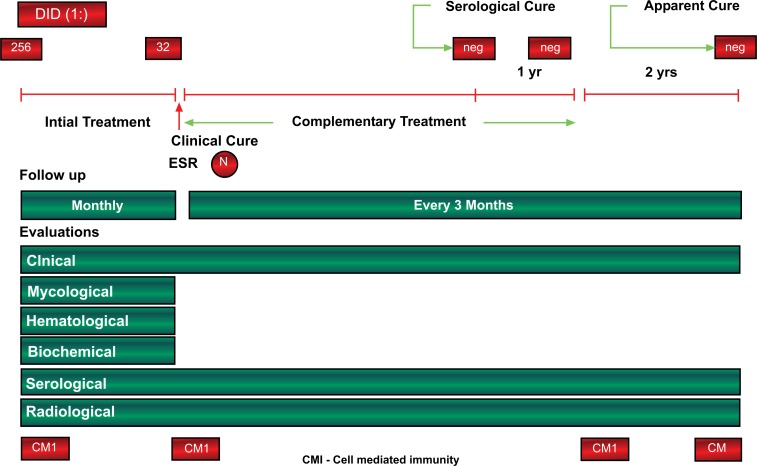
Schedule of treatment and follow-up of patients with paracoccidioidomycosis. DID - serological evaluation performed by the double agar gel immunodiffusion test, as the inverse of the dilution. CMI – evaluation of the cell mediated immunity.

**Fig. (10):**
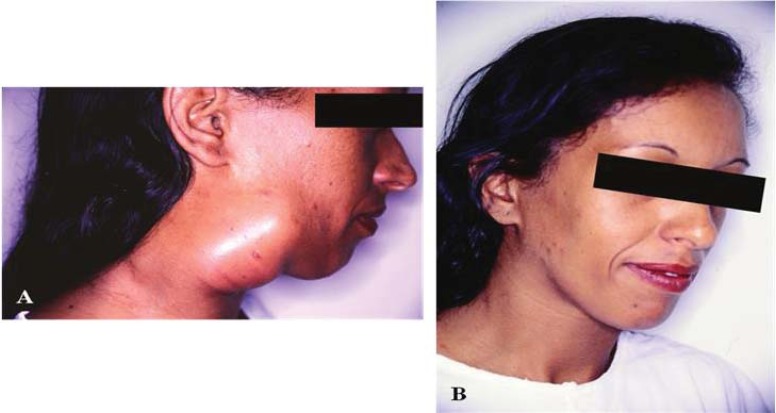
Patient with the acute/subacute form of paracoccidioidomycosis. A – lymph-node enlargement of the suppurative type before treatmnent; B – the same patient after treatment with itraconazole.

**Table 1:** Prevalence (percentage) of organs involved in patients with paracoccidioidomycosis. Study of autopsies.

	**Benaim-Pinto *et al*.** **1961** **N=50**	**Del Negro** **1961** **N=56**	**Brass *et al*.** **1969** **N=36**	**Dillon** **1972** **N=14**	**Salfeder** **1969** **N=11**	**Defaveri *et al*.** **2002** **N=13 N=40**	
**Organs**						**Acute**	**Chronic**
**Lungs**	69.6	67.8	75.0	42.0	100.0	100.0	97.5
**Limph nodes**	67.7	64.3	33.0	28.0	72.7	**-**	50.0
**Oral mucosa, pharynx, larynx**	55.6	41.1	40.0	**-**	18.2	**-**	70.0
**Adrenals**	56.7	48.2	80.0	57.0	36.3	75.0	63.0
**Central nervous system**	2.2	12.5	**-**	21.0	**-**	**-**	**-**
**Liver**	29.0	37.5	27.0	21.0	45.5	100.0	**-**
**Spleen**	17.6	39.3	2.7	21.0	54.5	**-**	**-**
**Skin**	31.3	39.3	2.7	64.0	-	**-**	**-**
**Kidneys**	6.2	19.6	8.3	14.0	9.1	**-**	**-**
**Bowels**	23.4	28.4	2.7	**-**	**-**	**-**	**-**
**Bone marrow**	**-**	**-**	**-**	**-**	**-**	75.0	**-**
**Heart**	2.0	-	2.7	7.0	9.1	**-**	**-**

Benaim-Pinto H. Mycopathologia 1961; 15: 90 – 114. Del Negro G. [tese]. Faculdade de Medicina da Universidade de São Paulo, São Paulo (SP); 1961. Brass K. Mycopathologia et Mycologia Applicata 1969; 37: 119 – 138. Dillon NL. [tese]. Faculdade de Medicina da Universidade de São Paulo, São Paulo (SP); 1972. Salfelder K, Doehnert G, Doehnert H-R. Virchows Arch Abt. A Path Anat 1969; 348: 51 – 76. Defaveri J, Joaquim A. Ann Rev Biomed Sci 2002 (special issue): 111.

**Table 2:** Immunological profiles among paracoccidioidomycosis patients with acute/subacute (FA) and chronic (FC) forms and healthy individuals (controls) according to the production of cytokines by different subpopulations of leukocytes in response to *P. brasiliensis* antigen (*Pb*Ag).

**Leucocyte Subsets** **Profile (Cytokines)**	**No Stimulus**			***Pb*Ag**			**References**
	**Control**	**FA**	**FC**	**Control**	**FA**	**FC**	
PBMC							
**Th1**(IFN-γ, IL-2, IL-12)	+	+	+	+++	+	++	[171, 184, 185]
**Th2/Th9**(IL-4, IL-5, IL-9)	+	+	+	+	+++	+	[171, 185, 186]
**Treg**(TGF-β1, IL-10)	+	+++	++	++	+++	+++	[171, 184, 186]
**Th17/Th22**(IL-17, IL-22)	0	0	++	NR	NR	NR	[171]
**Pro-inflammatory**(TNF-α, IL-6)	++	+	++	NR	NR	NR	[186]
CD4							
**Th1**(IFN-γ, IL-2, TNF-α)	+++	+	++	NR	NR	NR	[187]
CD8							
**Th1**(IFN-γ, IL-2, TNF-α)	++	+	+	NR	NR	NR	[187]
Monocytes							
**Pro-inflammatory**(TNF-α, IL-6, IL1-β, IL-12, MIP-1α, H_2_0_2_)	+	+	++	++	NR	+++	[178, 187-189]
**Anti-inflammatory**(IL-10, TGFβ1)	+	++	++	++	NR	+++	[178, 187, 189]
**Pro-fibrotic**(FGFb, TGFβ1)	++	NR	++	+	NR	++	[178, 189]
**Alveolar macrophages**Pro-inflammatory (H_2_0_2_)	+	NR	+++	NR	NR	NR	[188]

PBMC = peripheral blood mononuclear cells; NR = not realized; 0 = absent; + = mild; ++ = moderate; +++ = intense

**Table 3:** Prevalence and intensity of the altered biochemical variables in 200 paracocidioidomycosis patients with active disease, before treatment with antifungal compounds. Comparison between the acute/subacute and the chronic form.

	**Prevalence (%)**				**Intensity**		
**Variables**	**AF+CF**	**AF**	**CF**	***p* value**	**AF**	**CF**	***p* value**
**AST [a]**	12.7	21.7	9.6	0.05<p<0.10	2.5±1.6	1.5±0.6	0.13
**ALT [a]**	18.2	23.9	16.3	0.10	2.8±1.6	1.7±0.6	0.06
**TB [a]**	6.2	8.7	5.3	0.20	6.4±4.6	1.9±1.2	0.07
**CB [a]**	12.0	21.7	8.5	0.05	10.5±14.3	5.0±5.1	0.22
**ALP [a]**	26.0	50.0	17.8	<0.001	2.8±2.0	1.4±0.4	0.001
**γ-GT [a]**	32.6	47.8	24.7	0.02	5.2±6.6	2.7±2.9	0.02
**Triglycerides [a]**	10.7	4.4	12.7	0.12	2.20±1.70	1.37±0.50	…
**Glucose [a]**	14.9	15.2	14.8	0.94	1.23±0.32	1.32±0.41	0.59
**Glucose [d]**	4.3	6.5	3.5	0.39	0.70±0.19	0.75±0.34	…
**Cholesterol [a]**	33.7	18.8	38.7	0.01	1.26±0.37	1.16±0.17	0.18
**Cholesterol [d]**	66.3	81.3	61.3	0.01	0.72±0.13	0.81±0.13	≤0.001
**Total lipids [d]**	8.8	23.5	3.9	0.01	0.82±0.07	0.82±0.12	…
**Calcium [a]**	8.7	9.3	8.5	0.87	1.04±0.04	1.04±0.04	…
**Calcium [d]**	7.1	11.6	5.7	0.18	0.95±0.04	0.95±0.03	0.71
**Uric acid [d]**	11.9	3.0	15.4	0.07	…	1.13±0.12	…
**Phosphorus [a]**	14.3	14.3	14.3	1.0	1.06±0.02	1.18±0.18	0.18
**Phosphorus [d]**	4.0	40.0	37.5	0.79	0.81±0.10	0.86±0.10	0.16
**α_1_- glycoprotein [a]**	37.8	38.9	37.5	0.92	1.60±0.51	1.39±0.37	0.24
**Total protein [a]**	31.6	48.9	25.7	0.003	1.08±0.08	1.10±0.30	0.72
**Total protein [d]**	4.8	4.3	5.0	0.84	0.85±0.03	0.80±0.29	…
**Albumin[d]**	24.2	30.4	20.2	0.26	0.74±0.21	0.83±0.12	≤0.001
**Globulins [a]**	73.0	84.4	69.3	0.046	1.44±0.31	1.23±0.20	≤0.001
**Mucoproteins [a]**	52.7	56.5	51.5	0.68	1.60±0.50	1.58±4.3	0.91

**Table 4:** Parameters of accuracy of serological tests for diagnosis of paracoccidioidomycosis. Detection of serum antibodies. Cross reactions with other diseases.

**Accuracy Parameters**									
**Method**	**Antigen**	**S**	E*	**PPV**	**NPV**	**A**	**PLR**	**NLR**	**Ref.**
**CF^a^**	FL	93.0	96.7	93.0	96.7	95.5	31.0	0.06	^336^
**CF^a^**	FL	69.5	100.0	100.0	100.0	84.9	34.5	0.30	^337^
**RFC**	Po	…	…	…	…	…	…	…	^339^
**DID**	FL	76.2	100.0	100.0	90.0	93.0	38.0	0.23	^23^
**CIE**	FL	77.0	95.0	…	…	94.0	…	…	^341^
**LA**	CrudeEthanol	69.660.9	80.083.0	61.562.2	85.182.2	76.776.0	3.453.52	0.370.47	^342^
**LA**	Crude	84.0	100.0	100.0	71.0	88.0	21.0	0.15	^343^
**IFA**	FL	98.0	82.0	97.0	95.0	94.0	12.25	0.01	^346^
**ELISA^b^**	FL	100.0	100.0	100.0	100.0	100.0	125.0	0.014	^348^
**ELISA^c^**	CT	66.0	95.0	94.0	68.0	78.0	13.2	0.35	^349^
**ELISA**	gp43	100.0	…	…	…	…	…	…	^350^
**Dot-blot**	p 27 recombinant	100.0	100.0	100.0	100.0	100.0	11.1	0.09	^352^
**Dot-blot**	gp43	100.0	…	…	…	…	…	…	^353^
**Dot-ELISA**	FL	91.0	95.4	96.0	98.2	93.0	…	…	^354^
**IB**	gp43gp70	100.096.0	......	......	......	......	......	......	^355^
**IDD**	CFA-Plexo-PlTCA-Pl	10058.817.6	100.0100.0100.0	100.0100.0100.0	100.089.522.0	.........	.........	.........	^356^
**ELISA^d^**	FL	96.096.9	95.095.1	95.095.0	96.097.0	95.596.0	19.231.67	0.040.05	^368^
Cross reactions**									
**Method**	**Antigen**	**TBC**	**HST**	**ASP**	**CRC**	**LSZ**	**CCD**	**ESP**	**^Ref.^**
**CF^a^**	FL	3.3	23.5	3.8	…	…	…	…	^336^
**CF^a^**	FL	0.0	14.2	0.0	0.0	0.0	…	…	^337^
**RFC**	Po	…	52.6	…	60.0	…	0.0	65.2	^339^
**DID**	FL	…	0.0	…	…	…	0.0	0.0	^23^
**CIE**	FL	…	…	…	…	…	…	…	^341^
**LA**	CrudeEthanol	17.032.0	46.934.4	47.415.8	……	……	……	……	^342^
**LA**	Crude	…	27.2	26.7	…	…	…	…	^343^
**IFA**	FL	8.0	34.0	…	…	…	2.0	…	^346^
**ELISA^b^**	FL	33.0	55.0	22.0	50.0	55.0	…	0.0	^348^
**ELISA^c^**	CT	…	36.0	…	0.0	…	0.0	…	^349^
**ELISA**	gp43	…	53.1	…	…	30.7	…	…	^350^
**Dot-blot**	p 27 recombinant	0.0	0.0	10.0	0.0	…	0.0	0.0	^352^
**Dot-blot**	gp43	…	0.0	0.0	0.0	31.3	…	…	^353^
**Dot-ELISA**	FL	…	…	…	…	…	…	…	^354^
**IB**	gp43gp70	……	100.042.5	0.00.0	……	0.00.0	……	……	^355^
**IDD**	CFA-Plexo-PlTCA-Pl	………	………	………	………	………	………	………	^356^
**ELISA^d^**	FL	41.420.7	35.834.8	43.528.5	……	……	……	……	^368^

*Evaluated in healthy donors; ** Evaluated in patients with microbiologically confirmed active disease; DID: double agar gel immunodiffusion test; ELISA: enzyme-linked immunosorbent assay; CF: complement fixation; CIE: counterimmunoelectrophoresis; IFA: indirect immunofluorescence assay; LA: latex agglutination test; IB: immunoblotting assay; cut-off - ^a^: ≥1:8;^b^: 1:80; ^c^: 2,0; ^d^: 0.710 and 0.850; FL: culture filtrate antigen yeast phase; CT: cytoplasmic antigen; Po: polysaccharide antigen; p 27: recombinant proteinEthanol: antigen treated with ethanol; Exo-Pl: crude exoantigen of *P. lutzii;* CFA-Pl: cell free antigen of *P. lutzii;* TCA-Pl: crude exoantigen of *P. lutzii* precipitated with trichloroacetic acid) S: sensitivity, E: specificity; PPV / NPV: positive and negative predictive values; A: accuracyPLR: positive likelihood ratio; NLR: negative likelihood ratio; TBC: tuberculosis; HST: histoplasmosis; ASP: aspegillosis; CRC: cryptococcosis; LSZ: lacaziosis; CCD: coccidioidomycosis; ESP: esporotrichosis.

**Table 5:** Parameters of accuracy of serological tests for diagnosis of paracoccidioidomycosis. Detection of serum antigens. Cross reactions with other diseases.

**Accuracy parameters**									
**Method**	**Monoclonal antibodies**	**S**	E*	**PPV**	**NPV**	**A**	**PLR**	**NLR**	**Ref.**
***Inhi*-ELISA^a^**	FL	80.0	100.0	100.0	69.0	86.0	20.0	0.2	^357^
***Inhi*-ELISA^b^**	gp43	96.2	96.7	96.2	96.7	96.5	32.0	0.03	^358^
***Inhi*-ELISA^c^**	gp43 / gp70	90.9	100.0	100.0	96.7	97.5	30.0	0.09	^360^
Cross reactions**									
**Method**	**Monoclonal antibodies**	**TBC**	**HST**	**ASP**	**CRC**	**LSZ**	**CCD**	**ESP**	**Ref.**
***Inhi*-ELISA^a^**	FL	55.5	40.0	100.0	10.0	…	…	11.1	^357^
***Inhi*-ELISA^b^**	gp43	…	0.0	…	0.0	…	…	…	^358^
***Inhi*-ELISA^c^**	gp43 / gp70	…	…	…	…	…	…	…	^360^

*Evaluated in healthy donors; ** Evaluated in patients with microbiologically confirmed active disease; *Inhi*-ELISA: *Inhibition*-ELISA; cut-off - ^a^: >3.3µg/mL; ^b^:>1.353µg/mL; ^c^: 0.23g/mL for gp43 and 0.97g/mL for gp70; FL: culture filtrate antigen yeast phase; S: sensitivity, E: specificity; PPV / NPV: positive and negative predictive values; A: accuracy; PLR: positive likelihood ratio; NLR: negative likelihood ratio; TBC: tuberculosis; HST: histoplasmosis; ASP: aspegillosis; CRC: cryptococcosis; LSZ: lacaziosis; CCD: coccidioidomycosis; ESP: esporotrichosis.

**Table 6:** Antifungal compounds indicated in the treatment of paracoccidioidomycosis.

**Antifungal compounds**	**Route of administration**	**Daily doses**	**Intervals among doses (hours)**	**Adiministration** **with food**	Concentration in CSF (%)**
**Amphotericin B deoxycollate**	IV	increasing* mdd = 50 mg	48	..	Mínima
**Sulfadiazine**	PO	100 mg / kg mdd = 4,0 g [150mg / kg ou 4g / m^2^]	6	indifferent	40 a 60
**SMX-TMP**	IV, PO	2.400 mg / 480 mg [8-10mg / kg TMP]	12	indifferent	SMX=40 TMP=50
**Ketoconazole**	PO	400 mg [5-8mg / kg]	24	no 2 h before or 2 h after	< 10
**Itraconazole**	PO	200 mg [5mg / kg]	12 or 24	yes	< 1
**Fluconazole**	IV, PO	400 mg [12mg / kg]	12 or 24	indifferent	> 70
**Voriconazole**	IV, PO	400 mg*** [8mg / kg]	12	no 2 h before or 2 h after	> 50

SMX-TMP – trimethoprin-sulfametoxazol combination TMP – trimetoprima IV- intravenous PO – per os mdd – maximum daily doses

*administration in alternate days; increasing doses from 5.0 mg up to 1.0 mg/kg every administration; maximum dosis of 50mg.

** Cerebrospinal fluid concentration: porcentage of the plasmatic level

*** 400 mg 12/12h only in the first day

[ ] – pediatric dosage
